# Dramatic differences in carbon dioxide adsorption and initial steps of reduction between silver and copper

**DOI:** 10.1038/s41467-019-09846-y

**Published:** 2019-04-23

**Authors:** Yifan Ye, Hao Yang, Jin Qian, Hongyang Su, Kyung-Jae Lee, Tao Cheng, Hai Xiao, Junko Yano, William A. Goddard, Ethan J. Crumlin

**Affiliations:** 1 Joint Center for Artificial Photosynthesis, Lawrence Berkeley National Laboratory, Berkeley, CA 94720 USA; 2 Advanced Light Source, Lawrence Berkeley National Laboratory, Berkeley, CA 94720 USA; 3 Chemical Sciences Division, Lawrence Berkeley National Laboratory, Berkeley, CA 94720 USA; 4 Materials and Process Simulation Center, California Institute of Technology, Pasadena, CA 91125 USA; 5 Hefei National Laboratory for Physical Sciences at the Microscale, University of Science and Technology of China, Hefei, Anhui 230026 China; 6 Department of Physics and Photon Science, Gwangju Institute of Science and Technology (GIST), Gwangju, 500-712 South Korea; 7 Joint Center for Artificial Photosynthesis, California Institute of Technology, Pasadena, CA 91125 USA; 8 Molecular Biophysics and Integrated Bioimaging Division, Lawrence Berkeley National Laboratory, Berkeley, CA 94720 USA

**Keywords:** Catalytic mechanisms, Surface spectroscopy, Reaction mechanisms, Materials for energy and catalysis

## Abstract

Converting carbon dioxide (CO_2_) into liquid fuels and synthesis gas is a world-wide priority. But there is no experimental information on the initial atomic level events for CO_2_ electroreduction on the metal catalysts to provide the basis for developing improved catalysts. Here we combine ambient pressure X-ray photoelectron spectroscopy with quantum mechanics to examine the processes as Ag is exposed to CO_2_ both alone and in the presence of H_2_O at 298 K. We find that CO_2_ reacts with surface O on Ag to form a chemisorbed species (O = CO_2_^δ−^). Adding H_2_O and CO_2_ then leads to up to four water attaching on O = CO_2_^δ−^ and two water attaching on chemisorbed (*b-*)CO_2_. On Ag we find a much more favorable mechanism involving the O = CO_2_^δ−^ compared to that involving *b-*CO_2_ on Cu. Each metal surface modifies the gas-catalyst interactions, providing a basis for tuning CO_2_ adsorption behavior to facilitate selective product formations.

## Abstract (web summary)

The recycling of CO_2_ into storable chemicals is critical in order to mitigate climate change, although CO_2_’s inert nature has limited the reduction’s mechanistic considerations. Here, authors pair in-situ spectroscopy with quantum mechanics to elucidate CO_2_ adsorption on copper and silver surfaces.

## Introduction

In order to close the anthropogenic carbon circle while creating a sustainable hydrocarbon-based energy cycle, it is essential to discover new electrocatalysts that can efficiently convert carbon dioxide (CO_2_) into liquid fuels and feedstock chemicals^1–7^. However, CO_2_ is highly inert, making the CO_2_ reduction reaction (CO_2_RR) unfavorable thermodynamically. High throughput experimental and computational screening methods have been employed to discover new advanced CO_2_ reduction catalysts but these approaches are based on preconceived notions of the reaction mechanisms and have not produced dramatic successes^8–11^. To accelerate progress we believe that it is essential to develop a complete atomistic understanding of how CO_2_ interacts with and is transformed by the catalyst surfaces to provide guidance to design the catalyst to selectively tune the mechanisms for adsorption and activation.

Electrocatalysts such as Au, Ag, Zn, Pd, and Ga are known to yield mixtures of CO and H_2_ at varying ratios depending on the applied voltage^12–16^, with Ag exhibiting particularly high activity and selectivity to CO vs. H_2_. We sought to obtain a comprehensive understanding of how CO_2_ and H_2_O molecules adsorb on the Ag surface and interact to initiate CO_2_ dissociation and subsequent product formation.

Here we report our findings combining in-situ ambient pressure X-ray photoelectron spectroscopy (APXPS) with quantum mechanics (QM), which leads to unexpected and exciting findings for CO_2_ surface adsorption on Ag surface that are quite different than observed previously for Cu surfaces. We find that physisorbed linear (*l-*) and chemisorbed bent (*b-*) CO_2_ are not stable on pure Ag (111) surface, but rather gaseous (*g-*) CO_2_ reacts with on-top surface oxygen (O) atoms on Ag to form a chemisorbed species (O = CO_2_^δ−^). This surface species stabilizes up to four adsorbed H_2_O, through forming hydrogen bonds (HBs). We also find that a pair of surface H_2_O stabilize *b-*CO_2_ on the Ag by forming two HBs between the H_2_O_ads_ and CO_2_. The QM results and experimental observations suggest that the (O = CO_2_^δ−^)-(H_2_O)_n_ clusters are the main surface adsorbates with CO_2_ and H_2_O co-adsorption. On Ag we find a very different and more favorable mechanism of activating CO_2_, involving the O = CO_2_^δ−^, compared to that involving *b-*CO_2_ on Cu. Ag and Cu surfaces differ in both the chemical speciation and the respective adsorption energies, operating entirely differently for the first step of activating CO_2_.

## Results

### Dramatic differences in CO_2_ adsorption between Ag and Cu

**Fig. 1:**
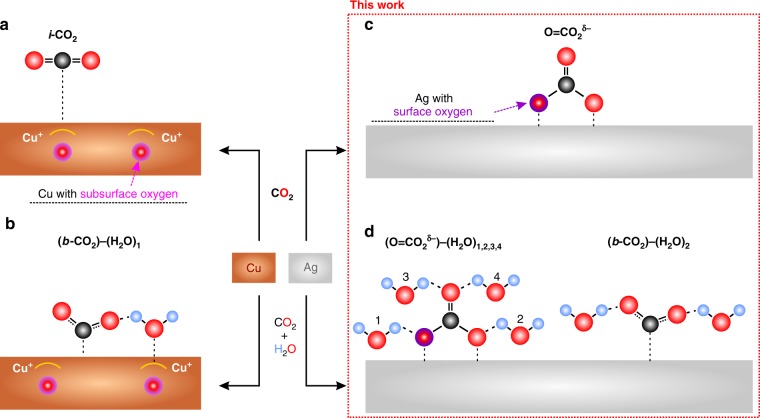
Overview of surface adsorptions and reactions of CO_2_ on Cu and Ag surfaces under various conditions. **a**, **b** We earlier reported CO_2_ adsorption on Cu (111) at 298 K both alone and in the presence of H_2_O. These studies concluded that subsurface oxygen leads to a surface Cu^+^ atom that stabilizes *l-*CO_2_ sufficiently strongly to be stable at 298 K and 0.7 Torr (**a**). In the presence of subsurface O, we found that H_2_O adsorbs preferentially to the Cu^+^ site while interacting sufficiently strongly with CO_2_ to stabilize the *b-*CO_2_, (through a hydrogen bond (**b**)) sufficiently to be stable at 298 K and 0.7 Torr total pressure. **c**, **d** Based on our new studies of adsorbed CO_2_ on the Ag surface alone and in the presence of H_2_O at 298 K. We find that *l-*CO_2_ is not stable on Ag surface even at CO_2_ pressure of 0.3 Torr at 298 K. However, CO_2_ reacts strongly with surface oxygen to form a carbonic acid like structure (**c**). This O = CO_2_^δ−^ species can stabilize one to four adsorbed H_2_O through hydrogen bonding (**d**). Furthermore, *b-*CO_2_ can also be stabilized by a pair of surface adsorbed H_2_O each forming a hydrogen bond with an O of *b-*CO_2_ (**d**)

For both Ag and Cu surfaces, we find that oxygen plays an essential role to induce reactions involving CO_2_ and H_2_O, but the consequences for each metal are dramatically different. The stability of surface and subsurface O in Ag and Cu surfaces are compared (see Supplementary Note 1; Supplementary Figs. 1 and 2; Supplementary Table 1), where we find that subsurface O, which stabilized both the *l-* and *b-* CO_2_ in the Cu system (Fig. 1a, b)^17,18^, is not stable on Ag; quantum mechanics (QM) finds that putting an O in an Ag subsurface site goes without a barrier to an on-top three-fold (η_3_) site (Supplementary Fig. 2). These on-top surface O atoms interact with gaseous (*g-*) CO_2_ to form a chemisorbed surface carbonic acid-like species in which two O on the C bind to adjacent Ag bridging sites, while the third O forms a C double bond (C = O) perpendicular to the surface. We denote this carbonic acid like adsorbate as O = CO_2_^δ−^ to indicate that the negative charge is on the two O binding to the Ag surface. Our combined QM and experimental results show that only O = CO_2_^δ−^ is stable prior to exposure to H_2_O. For Cu only unreactive *l-*CO_2_ is stable without H_2_O.

Adding H_2_O to the surface with O = CO_2_^δ−^ and *g-*CO_2_ leads to two kinds of structures stable at 298 K and the applied pressures, carbonic acid-like species attaching up to four water, (O = CO_2_^δ−^)-(H_2_O)_1-4_, and *b-*CO_2_ attaching two water. The observation of the surface cluster of (O = CO_2_^δ^−)-(H_2_O)_1-4_ is different from the previous understanding of CO_2_ on metal surfaces, which all involve (*b-*CO_2_)-(H_2_O)_n_ configurations.

### CO_2_ adsorption on Ag surfaces

The (111) surface is closest packed, making it the most favorable facet for Ag and Cu. Indeed experimental evidence shows that silver (and Cu) at high temperature exposes this facets^17,19,20^. Thus our simulations compare results on the Ag (111) surface with experimental observations on vacuum annealed polycrystalline Ag surface.

We started by carrying out QM studies to examine the stability of various surface adsorbates on pristine Ag surfaces, considering both *l-* and *b-* CO_2_. The optimized structure for *l-* and *b-* CO_2_ is found to be unfavorable with *E*_ads_ (QM electronic binding energies) = −0.15 eV and Δ*G* =  + 0.19 eV, and *E*_ads_ = + 0.77 eV and Δ*G* =  + 1.13 eV, respectively (Supplementary Fig. 2). These and all other ΔG values are from QM calculations including zero point energy, entropy, and specific heat to obtain ΔG at 298 K and at the pressure quoted.

### CO_2_ adsorption on oxygen treated Ag surfaces

We started the calculation by considering the possible promotion effect of sublayer oxygen that we found previously to stabilize CO_2_ adsorption on Cu surface. However, for Ag the QM finds that subsurface O minimizes to the O at the surface. In the presence of isolated surface O, we found that *l-*CO_2_ has Δ*E*_ads_ = −0.21 eV, but Δ*G* =  + 0.13 eV (Supplementary Note 2; Supplementary Fig. 2). Thus a pressure of ~30 Torr would be required to stabilize *l-*CO_2_ on the O/Ag surface at 298 K. This contrasts with observations for Cu, where subsurface O stabilized the adsorption of *l-*CO_2_ on Cu surface under 0.7 Torr CO_2_ partial pressure at 298 K^17^ (Fig. 1b). This attraction resulted from the subsurface O in a tetrahedral site inducing Cu^+^ character into the single Cu atom above it on the surface, which stabilized the *l-*CO_2_. This oxygen promotion effect is not observed for Ag because the O is chemisorbed on top of the Ag, which does not facilitate Ag oxidation (to Ag^+^)^19,21–25^. This contrasting result provides fresh insight into the tunability of CO_2_ adsorption on metal surfaces.

**Fig. 2:**
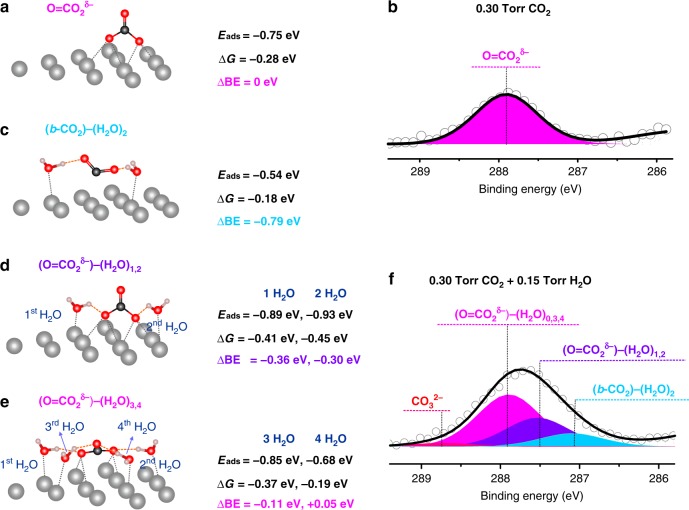
The QM predictions and experimental observations of Ag surface with CO_2_ adsorption alone and in the presence of H_2_O at 298 K. **a** Predicted structures for O = CO_2_^δ−^ on Ag surface. The O = CO_2_^δ−^ C 1s peak BE has been set as the reference point for subsequent experiments with H_2_O. **b** The C 1s APXPS spectra for Ag surfaces in the presence of 0.3 Torr CO_2_ at 298 K. One single C 1 s peak representing O = CO_2_^δ−^ was observed. **c** *b-*CO_2_ becomes stabilized by a pair of H_2_O_ad_ each forming a HB with an O of *b-*CO_2_, leading to ΔG of −0.18 eV with respect to desorbing H_2_O and CO_2_. **d**, **e** The adsorbed O = CO_2_^δ−^ species stabilizes one or two H_2_O_ad_ via HBs to the O_ad_ and two more water with HBs to the O_up_. O = CO_2_^δ−^ stabilizes the 1st, 2nd, 3rd, and 4th H_2_O on this site with ΔG of −0.41 eV, −0.45 eV, −0.37 eV, and −0.19 eV, respectively. **f** The C 1 s APXPS spectra and the peak deconvolution results for Ag surfaces in the presence of 0.3 Torr CO_2_ and 0.15 Torr H_2_O at 298 K. This deconvolution used the peak separations from the theory. The new surface adsorbates, (O = CO_2_^δ−^)-(H_2_O)_1,2_ and (*b-*CO_2_)-(H_2_O)_2_, are observed experimentally in the APXPS measurements, showing up as the two new peaks at 0.4 eV and 0.8 eV, lower than the O = CO_2_^δ−^ peak. The species (O = CO_2_^δ−^)–(H_2_O)_3,4_ do not lead to additional peaks, because they are located at position that overlaps with that of O = CO_2_^δ−^

We evaluated the stabilization of *b-*CO_2_ next to surface O_ad_ on Ag, but the QM minimizes to form a surface carbonic acid-like species (Supplementary Fig. 2) with a C = O_up_ double bond (1.222 Å) pointing up while the other two O bind to adjacent three fold Ag (111) sites with C-O lengths of 1.365 Å and 1.354 Å and O-Ag distances of 2.276 Å (Fig. 2a). This is not an ionic carbonate possessing three similar O atoms, as had been speculated previously^26–28^. The CO_2_ bonding energy to form surface O = CO_2_^δ−^ is Δ*E*_ads_ = −0.75 eV, Δ*G* = −0.28 eV. We denote this carbonic acid-like adsorbate as O = CO_2_^δ−^ to indicate that the negative charge is on the two O binding to the Ag surface. The total charge of O = CO_2_^δ−^ is −1.26e^−^ and charge on C is +1.46, leading to C 1s binding energy (BE) of −269.45 eV. The simulated BE value corresponds to 287.9 eV in the experimental observation (Fig. 2b). The configuration of the O = CO_2_^δ−^ illustrated in top view is shown in Supplementary Fig. 3. The properties of the surface O = CO_2_^δ−^ are summarized in Supplementary Note 3, and Supplementary Figs. 4 and 5. The simulated vibrational frequency data for O = CO_2_^δ−^ are summarized in Supplementary Table 2.

We also investigated structures with vertical and horizontal CO_3_ configurations on the Ag (111) surface (Supplementary Fig. 6). We find that the structure with one O bridging to the surface and two C–O bonds pointing up is not stable with *E*_ads_ = + 0.32 eV. This starting structure rotates to form the stable bidentate species. We also examined the stability of the horizontal CO_3_ configuration with three C–O bonds constrained to be parallel to the Ag surface. This configuration is not stable. The CO_2_ bonding energy to form this horizontal structure is Δ*E*_ads_ = −0.34 eV, Δ*G* =  + 0.13 eV. Moreover the adsorption of CO_2_ on the Ag (111) surface with an Ag vacancy induced by oxygen adsorption was examined and found to be unstable on this structure (Supplementary Fig. 7).

The adsorption states of CO_2_ on various Ag surfaces at 298 K were monitored by C 1 s APXPS. The pristine Ag surface shows no detectable carbon- and oxygen-based contamination (Supplementary Fig. 8), while dosing O_2_ under different experimental conditions results in various oxygen coverages on Ag surface, that we monitor via the changes of the O_ad_ peak intensity (the detailed characterizations of the surface are shown and discussed in Supplementary Note 4 and Supplementary Figs. 9 and 10).

We partition the C 1 s spectra obtained on clean and oxygen-covered Ag surfaces into two parts. First, high binding energy region from 286–290 eV, showing the surface adsorbate, O = CO_2_^δ−^ at 287.9 eV. O = CO_2_^δ−^ is the only stable species on the Ag surface when exposed solely to CO_2_ (no H_2_O is present), leading to a single C 1 s peak in the adsorbate signal region of the APXPS spectra (Fig. 2b and Supplementary Fig. 11). Second, low binding energy region from 282 eV to 286 eV represents the surface reaction products from possible reactive carbon compounds (e.g., unsaturated hydrocarbons) from the chamber. The chemical species can be assigned as atomic C or carbide (283.0 eV), sp^2^ C = C (284.2 eV), sp^3^ C–C (285.2 eV), and C–O(H) (286.0 eV)^29–32^ (Supplementary Fig. 12).

Formation of the carbonic acid-like species requires O_ad_, which can be constituted from O_2_ pre-dosing and CO_2_ self-decomposition prior to the CO_2_ adsorption. The experimental O 1 s spectra shown in Supplementary Fig. 9 provide insight to elucidate the surface chemistry. The two peaks that represent two O atoms attached to Ag surface and the single O atom in the C = O bond, were used to fit the spectra. The energy difference between these two peaks was set as 0.7 eV based on the QM results. This leads to 2:1 peak intensity ratio. Thus the peak fitting of the experimental data supports the QM results. By further comparing the C and O signals, we obtain that the C:O atomic ratio are 1:2.85, 1:3.13, and 1:2.97 for adsorbates on pristine and low and high oxygen covered Ag surfaces, which are all close to 1:3, providing another strong evidence of the formation of CO_3_^δ−^ structure on Ag surface.

**Fig. 3:**
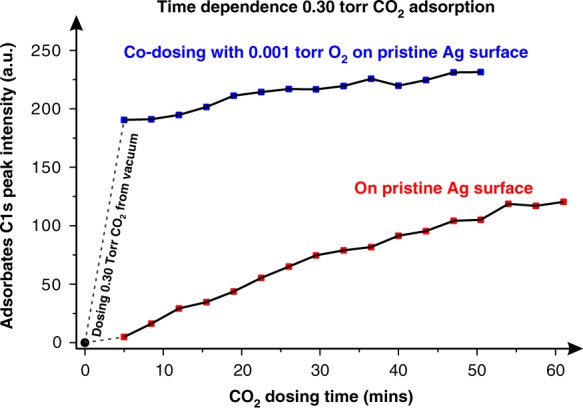
The C 1 s signal of surface adsorbate increase as a function of CO_2_ dosing time. The adsorbate signals for 0.3 Torr CO_2_ adsorption alone and in the presence of 0.001 Torr O_2_ were recorded at an increased dosing time, shown as red and blue points, respectively. A black line across the data point is used for the eye guidance. The peak intensity is the raw intensity without considering the signal decay due to introducing gases

Next, the adsorption of CO_2_ on pristine Ag surface both alone and at the presence of 0.001 Torr O_2_ at 298 K were investigated by recording the C 1 s peak intensity as a function of gas dosing time (Fig. 3). The first spectrum was recorded after dosing CO_2_ for 5 mins, which is the time period needed to reach 0.3 Torr pressure from the vacuum. In the case of the CO_2_ adsorption, the adsorbate peak is negligible in the first spectrum recorded after 5 mins of CO_2_ dosing, and it increases significantly as a function of increasing CO_2_ dosing time, finally, it reaches equilibrate state after 60 mins gas adsorption. Adding O_2_ with CO_2_, even a ratio of 1:300, significantly promots the process of CO_2_ adsorption on metallic Ag. The adsorbate signal is strong in the first spectrum, and it does not change dramatically as a function of the increasing dosing time. During this dynamic process, the O:C atomic ratio were calculated to be around 3:1, validating the surface adsorbate of CO_3_^δ−^ structure, as shown in Supplementary Fig. 13. The largely accelerated process for the surface to reach equilibrium by adding O_2_ is due to the formation of surface oxygen. Since CO_2_ adsorption on clean (non-oxygen pretreated) Ag surface requires a CO_2_ dissociation process prior to the formation of the final surface adsorbate, the dynamics of O = CO_2_^δ−^ formation on clean Ag surface is slower than that with the oxygen co-dosed.

It is well known that during ambient pressure exposure of CO_2_, possible residual reactive carbon compounds (e.g., unsaturated hydrocarbons) can be desorbed from the chamber. Thus, due to the slow surface reaction of CO_2_ on the clean Ag surface could lead to a larger possibility for the Ag surface to be exposed to unsaturated hydrocarbons that can lead to the formation of the sp^2^ carbon species. After the surface acquires surface O_ad_ (Supplementary Fig. 9) or is co-dosed with O_2_ (Fig. 3), CO_2_ can react directly on the surface to form O = CO_2_^δ−^. This suppresses surface carbon formation as evident in the decrease of the surface carbon (mainly the sp^2^ C = C^29–32^) C 1 s signals (Supplementary Fig. 12), resulting in more available surfaces sites to increase the amount of adsorbed O = CO_2_^δ−^ (Fig. 3 and Supplementary Fig. 11).

In addition, we made an estimate of the surface coverage by calculating the Ag and O atomic ratio, and the O = CO_2_^δ−^:Ag_suf_ ratios, which we found to be around 0.4:1, 0.6:1, and 0.7:1. This indicates that the reaction between surface O and Ag to form O = CO_2_^δ−^ happens at surface majority sites, justifying the use of the Ag (111) model in the this study.

### CO_2_ adsorption on Ag surfaces in the presence of H_2_O

The QM studies find that the *l*-CO_2_ configuration on Ag surface is not stable even considering the possible promotion effects of both O_ad_ and adsorbed water (H_2_O_ad_) (Supplementary Fig. 2). Adding H_2_O to the surface with O = CO_2_^δ−^ formed from *g-*CO_2_ leads to two groups of structures stable at 298 K and the applied pressures (Fig. 1d). First, a pair of surface H_2_O stabilizes *b-*CO_2_ on the Ag surface by forming two HBs between the H_2_O_ad_ and CO_2_ (Fig. 2c). Second, O = CO_2_^δ−^ can stabilize up to 4 H_2_O molecules through formation of HBs to the surface bonds of O = CO_2_^δ−^. The 1^st^ and 2^nd^ H_2_O_ad_ each forms a HB to one O_ad_ bonded to the surface (Fig. 2d), while adding the 3rd and 4th H_2_O force the C = O_up_ bond to rotate from being perpendicular to the surface to being nearly parallel to the surface, allowing the formation of HB from a 3rd and 4th surface H_2_O_ad_ to the two sp^2^ lone pairs on the C = O_up_ unit (Fig. 2e and Supplementary Figs. 3 and 4). From QM predictions, the 1st and 2nd H_2_O_ad_ shift the C 1s from −269.45 eV to −269.09 eV and −269.15 eV, while the 3rd and 4th H_2_O_ad_ shift the C 1s back to −269.34 eV and −269.50 eV, nearly the same BE’s as for no H_2_O_ad_ bonding (Fig. 2f and Supplementary Fig. 5). Considering that the O = CO_2_^δ−^ and surface water stabilize each other through HB, an increase in the surface adsorbate coverage when dosing CO_2_ in the presence of H_2_O is expected. This was experimentally observed as a dramatic adsorbate signal increase of C 1 s spectra compared to that from the adsorption of CO_2_ alone (Supplementary Figs. 11 and 12). Moreover, the simulated vibrational frequency data for (O = CO_2_^δ−^)−(H_2_O)_1-4_ and *b-*CO_2_−(H_2_O)_2_ are summarized in Supplementary Table 2.

### The tunability of CO_2_ adsorption on metal surfaces

**Fig. 4:**
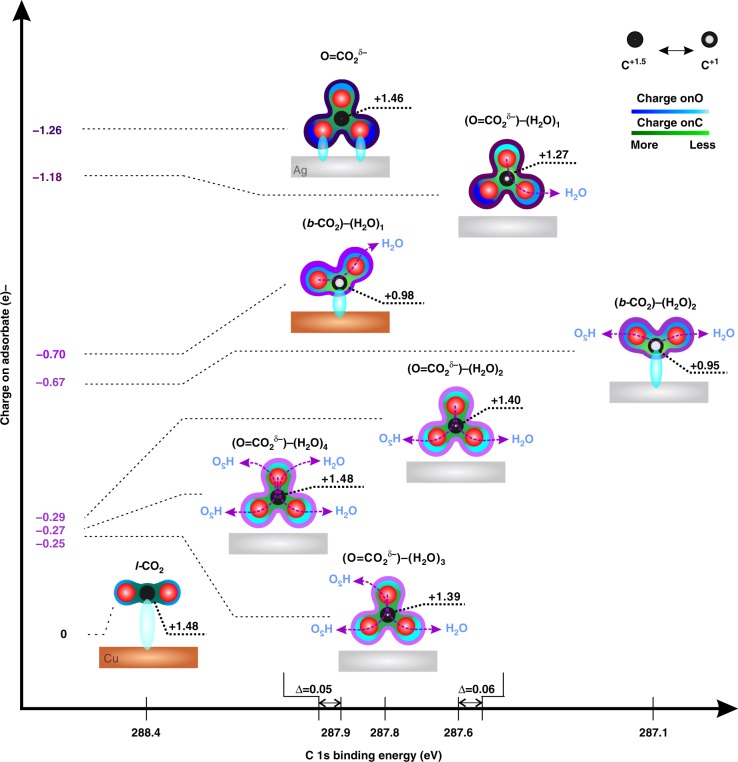
The electronic properties of various surface adsorbates on Ag and Cu. The charge distribution on the C, O and the adsorbates are summarized, with the corresponding C 1 s BE revisited. The various configurations of the adsorbates on the surface modify the charge transfer process, leading to different charge distribution on the adsorbates. Compared to *l-*CO_2_ (only observed on Cu surface), CO_2_ in the bent configuration exhibits extra charge accumulation. *b-*CO_2_ is stabilized on Ag only with two surface H_2_O but the charge distribution is similar to *b-*CO_2_ on Cu surface. However, their different distances to the Ag and Cu surface lead to different C 1 s peak BE’s. With the formation of the first two HBs to surface H_2_O, the total charge on O = CO_2_^δ−^ decreases, which decreases the C 1s BE by ~0.30 eV. But adding the 3rd and 4th H_2_O with HB to the C = O_up_ of the O = CO_2_^δ−^ increases the charge, shifting the BE back to 0.05 eV above the peak for no H_2_O. Thus the predicting C 1 s shifts and charge distribution on surface adsorbates are fully consistent with the experimental observed C 1 s BEs. These observed differences show the tunability of CO_2_ adsorption on the metal surfaces

Activating inert CO_2_ to *b-*CO_2_ requires both a change of the geometric molecular structure and accommodation of extra charge. For Cu, *b-*CO_2_ is stabilized by a subsurface O combined with a single surface adsorbed H_2_O_ad_ while for Ag it is stabilized by two adsorbed H_2_O_ad_. The *b-*CO_2_ with surface H_2_O configuration leads to a similar amount of charge transferred directly from the metal catalyst to the C for both Cu and Ag. Interestingly, the *b-*CO_2_ on Ag and Cu surfaces show similar charge distribution (calculated by performing Bader Charge Analysis on optimized structures^33–35^) but different C 1s binding energy peak positions. This may be ascribed to the increased final state screening effect of Cu on surface *b-*CO_2_ due to the smaller distance between the surface adsorbate and the metal substrate (2.55 Å for C–Ag vs. 1.69 Å for C-Cu)^36^. The direct Ag–C interaction in (*b-*CO_2_)−(H_2_O)_2_ leads to a −0.67e^−^ charge accumulating on the adsorbed CO_2_ molecule which is larger than the −0.3e^−^ for the O = CO_2_^δ−^ configuration (compared to O_ad_) (Fig. 4). Moreover, adding surface H_2_O leads to additional charge redistribution through the hydrogen bonding (Fig. 4). Attaching more water to O = CO_2_^δ−^ decreases the total charge on adsorbates, while the 1^st^ H_2_O decreases the charge on C atoms to +1.27 and the 2nd to 4th shift it back to +1.48, nearly the same as for no H_2_O (Fig. 4). The charge distribution on the various surface adsorbates are detailed in Supplementary Note 3.

This work highlights that the charge transfer configurations are responsible for the tunability of CO_2_ adsorption on the metal catalyst surface. These results suggest two modes for stabilizing adsorbed CO_2_. In the case of Cu, a subsurface O provided a positive Cu^+^ on the surface that stabilized water molecule sufficiently to stabilize *b-*CO_2_. This mechanism has been studied previously^37^.

For Ag there is no subsurface O, but the surface O_ad_ promotes the formation of surface carbonic acid-like species, O = CO_2_^δ−^, which leads to a very different reaction mechanism for Ag than for Cu. This new insight requires re-examining the subsequent steps of reactions to activate O = CO_2_^δ−^ to form products and how this depends on surface structure, solvent, pH, applied potential, the presence of anions and cations, and alloying with nonmetals (S, P, N, Cl) that might change the local charges and structures.

### Proposed CO_2_ reduction reaction pathway on Ag and Cu

**Fig. 5:**
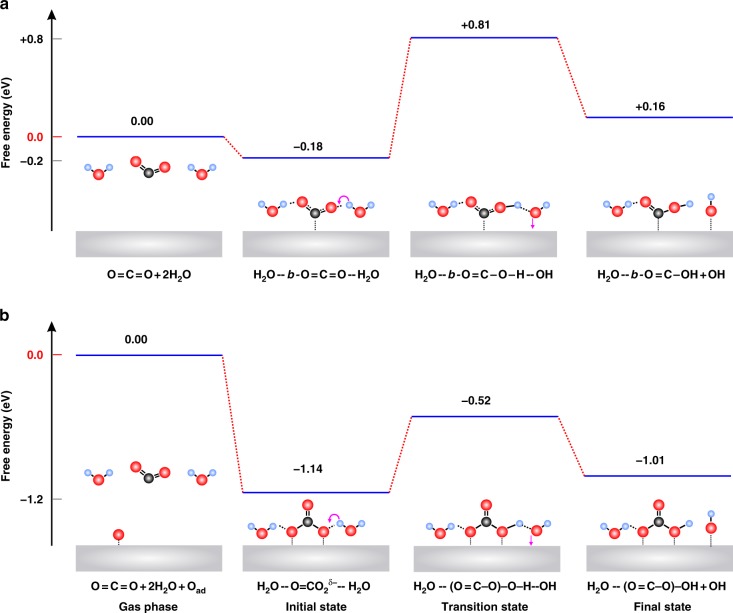
The QM predicted kinetic pathway for the CO_2_ hydrogenation process from full explicit solvent calculations. **a** The reaction pathway starting with *b-*CO_2_, the energy level of each step is referenced to *g-*CO_2_ and *g-*H_2_O; **b** The reaction pathway starting with O = CO_2_^δ−^, the energy level of each step is referenced to *g-*CO_2_, *g-*H_2_O and surface O_ad_. The first step was shown in Fig. 2, representing the stable adsorption configuration observed on the catalyst surface. The energy barrier information obtained from our climbing image nudged elastic band (NEB) calculations are detailed in Supplementary Fig. 14 We consider here the case of O = CO_2_^δ−^ with 2H_2_O to compare directly with *b-*CO_2_ + 2H_2_O

The CO_2_ adsorption on Ag contrasts dramatically from the results on Cu (Supplementary Table 3) providing possible explanations for why these metal catalysts have very different CO_2_ reduction performances. For Cu our full explicit solvent QM calculations for the initial step of CO_2_ to CO found that hydrogen bonding with the explicit solvent forms a similar *b-*CO_2_ stabilized by two surface H_2_O^37^. In that case, the next step is for one of these two H_2_O molecules to transfer an H to form the HOCO intermediate plus OH_ad_ and then a second surface H_2_O transfers an H to the OH of HOCO to form H_2_O plus OH_ad_, leading to CO_ad_, (this general reaction pathway is depicted in Fig. 5a).

For Ag with (*b-*CO_2_)−(H_2_O)_n_, Fig. 5a shows that the QM predicted free energy barrier is 0.99 eV on Ag for protonating the complex of *b-*CO_2_ with two H_2_O to form HOCO* plus OH* and H_2_O (Supplementary Fig. 14), leading to a total barrier of hydrogenation of CO_2_ to HOCO* of (−0.18) + (0.99) = 0.81 eV (Fig. 5a). This energy barrier is comparable to that on Cu, which is 0.80 eV^37^.

Surprisingly for Ag with (O = CO_2_^δ−^)-(H_2_O)_n_ clusters we find a different mechanism that is significantly more favorable. The discovery that (O = CO_2_^δ−^)−(H_2_O)_n_ is a stable surface cluster is most unprecedented, differing dramatically from our previous understanding of CO_2_ on a metal surface, which essentially all involve (*b-*CO_2_)−(H_2_O)_n_ configurations^38–42^.

We used QM to discover the mechanism of activation for the carbonic acid-like species on Ag. We find that the first step is for the H_2_O hydrogen bonded to the surface O to transfer an H to form the (C = O)(O)(OH) intermediate plus OH_ad_, as shown in Fig. 5b. The QM energy barrier is 0.62 eV, which is dramatically lower than the value of 0.80 eV for Cu, perhaps explaining the faster rate for Ag. Thus the barrier of hydrogenation of CO_2_ to OCOOH* of (−0.28) + (−0.41) + (−0.45) + (0.62) = −0.52 eV (Fig. 5b and Supplementary Fig. 14). This energy barrier is much smaller than for Cu. In particular, it is important to note that the energy levels of all the reaction steps starting with O = CO_2_^δ−^ are negative. This suggests that we might be able to see this reaction in APXPS by simply increasing the temperature. These results predict that the most energetically favorable reduction reaction pathway to hydrogenate CO_2_ to HOCO* involves the O = CO_2_^δ−^ configuration present only on Ag surface. This process is unprecedented and has never even been previously speculated. This result raises numerous questions about subsequent steps that will drive many new experimental and theoretical studies to determine the implications. Future studies will include the operando spectroscopic characterizations of these adsorbates under external potentials, and we will predict the Tafel slope to compare with previous experimental observations and to gain more insights into the new mechanism.

## Discussion

Our studies have established a comprehensive but totally new picture of the first steps of CO_2_ activation on Ag. The dramatic differences with Cu show how interactions between adsorbate and catalyst can be altered by tuning the charge transfer between them through changing the adsorption sites, configuration, and by introducing surface co-dosing adsorbates. These findings provide fresh insights about CO_2_ adsorption species and the initial steps of CO_2_ reduction mechanism on Ag surfaces. It is dramatically different from those on Cu surfaces, where *l-*CO_2_ leads to *b-*CO_2_ and then directly to CO_2_ reduction^32^.

Using synergistic experimental and theoretical analyses, we show that Cu and Ag operate entirely differently for the first step of activating CO_2_, even though the product CO is the same. We find that surface O (from O_2_ pre-dosing and CO_2_ self-decomposition) interacts with *g-*CO_2_ to form a carbonic acid like intermediate O = CO_2_^δ−^, the only stable species on Ag surface (exposed to CO_2_ only). Adding H_2_O and CO_2_ then leads to attaching up to four water on O = CO_2_^δ−^. In addition, two water stabilize *b-*CO_2_ on the Ag surface, which for Cu is the intermediate on the way to forming CO. On Ag we find a very different and much more favorable mechanism involving the O = CO_2_^δ−^, one that has not been suggested or observed previously. This raises numerous questions about the subsequent steps that could motivate the exploration of new chemistries.

These studies emphasize the power from combining BE, vibrational frequency, APXPS with QM for discovering the fundamentals underlying CO_2_ reduction. These unexpected findings will stimulate new thinking about the CO_2_ reduction reactions on metal surfaces, suggesting that stabilization of various surface adsorption configurations can be controlled through additives or alloying along with externally applied potentials to control the reaction processes.

## Methods

### QM calculations

All calculations were carried out with the Vienna Ab initio Simulation Package (VASP)^43^. We established that an energy cutoff of 500 eV leads to converged forces. The K-point sampling was chosen to be 3 × 3 × 1. All calculations include spin-polarization. We used the Perdew-Burke-Ernzerhof (PBE) flavor of Density Functional theory (DFT), including the D3 (Becke Johnson)^44^ empirical corrections for long range London dispersion (van der Waals attraction)^45^.

The PBE-D3(BJ) level of DFT leads to a calculated lattice parameter of *a* = 4.012 Å for the bulk Ag structure at 0 K, slightly smaller than the experimental value 4.085 Å at 298 K^46^. We used experimental lattice parameter 4.085 Å to construct a two-dimensional periodic slab with four layers of Ag atoms each of which consists of a (4 × 4) unit cell (16 surface Ag per cell). We include 15 Å of vacuum in the *z* direction to minimize possible interactions between the replicated cells. The top two layers are relaxed while the bottom layers are kept fixed.

This level of QM has been validated recently for several systems. Thus references carried out systematic studies for the oxygen reduction reaction (ORR, O_2_ + protons → H_2_O) on Pt (111) using the same PBE-D3 level as in this paper^47^. Including 5 layers of explicit solvent in QM metadynamics on all reaction steps, comparisons could be made to experimental activation barriers for two values of the external potential. In both cases the calculated activation barriers were within 0.05 eV of the experiment^48–51^.

Previous calculations for the CO_2_ reduction reaction on Cu (100) using the same level of theory obtain an activation energy within 0.05 eV of experiment. This same level of theory has also led to similar accuracy for the oxygen evolution reaction on IrO_2_ and for onset potentials on Cu (111)^52,53^.

Calculations for the gas phase molecules used the PBE functional (as implemented in Jaguar) with the D3 empirical correction for London dispersion^54^. To obtain the total free energy, G = H−TS, for the gas molecules at temperature T, we add to the DFT electronic energy (E), the zero-point energy (ZPE) from the vibrational levels (described as simple harmonic oscillators), and the specific heat corrections in the enthalpy from 0 to T. The entropy (S), as a sum of vibrational, rotational and translational contributions, are evaluated from the same levels. To correct the free energy for pressure, we assume an ideal gas and add RT × ln(*P*_2_/*P*_1_) with a reference pressure of *P* = 1 atm. For example, CO_2_ gas at room temperature and 1 atm would have a free energy correction of −0.25 eV, including ZPE (0.32 eV), translational entropy contribution (−0.42 eV), rotational entropy contribution (−0.15 eV) and almost negligible vibrational entropy contribution (−0.003 eV). All calculations assume the current experimental condition: *P*(CO_2_) = 0.3 Torr, and *P*(H_2_O) = 0.15 Torr.

After the gas molecules adsorbed on the metal surface, their rotational and translational degrees of freedom are reduced to vibrational modes. The vibrational frequencies for surface adsorbents are calculated by allowing the adsorbed molecules and the top layer of metal to relax, with the bottom layers fixed. For these phonon calculations we used 10^−6^ eV energy convergence threshold to obtain reliable phonon frequencies (no negative eigenvalues.) To obtain the Free energy, G = H−TS, for the various equilibrium configurations, we used density functional perturbation theory (DFPT) to calculate the phonon density of states, which was used to calculate the ZPE, the temperature correction to the enthalpy, and the vibrational contributions to the entropy.

There are two ways of calculating the change in core level energies implemented in VASP^43^. The simpler option (ICORELEVEL = 1) calculates the core levels in the initial state approximation, which involves recalculating the KS eigenvalues of the core states after a self-consistent calculation of the valence charge density. The second option (ICORELEVEL = 2) is more involved. In this case, electrons are removed from the core and placed into the valence. Our previous studies found that the ICORELEVEL = 1 leads to relative binding energy shift in good agreement with experimental XPS^17^.

### In-situ ambient pressure X-ray photoelectron spectroscopy measurements

Ambient pressure XPS measurements were performed at Beamline 9.3.2 of the Advanced Light Source, Lawrence Berkeley National Laboratory^55^. The beamline has station consisted of a load lock chamber with base pressure of ~5 × 10^−8^ Torr for sample loading; a preparation chamber with base pressure of ~1 × 10^−9^ Torr for sample preparation, and a main chamber for sample characterization under ambient pressure condition. The beamline provides beams with a photon energy range of 200–800 eV.

The pristine Ag surface was in-situ prepared in the vacuum chamber by repeated argon sputtering (2 keV, 60 min) and vacuum annealing (900 K, 60 min), leading to a clean surface with no detectable carbon- and oxygen- based contamination. The oxygen covered Ag surfaces were prepared by annealing the samples at 430 K at 0.04 Torr O_2_ for 5 min, and 0.06 Torr O_2_ for 15 min, respectively.

During the APXPS measurements performed at 298 K, CO_2_ partial pressure was kept at 0.3 Torr for CO_2_ adsorption, whereas the total pressure was kept at 0.45 Torr with 0.3 Torr CO_2_ and 0.15 Torr H_2_O. The purities of the dosing gases (CO_2_, H_2_O) were in-situ monitored by a conventional quadrupole mass spectrometer to ensure no additional gas cross-contamination (especially, the CO and H_2_ gases).

The XPS spectra were collected at an incident photon energy of 670 eV, in the following order: a low-resolution survey with a binding energy of 600 eV to –10 eV, then high-resolution scans of O 1 s, C 1 s and valence band. The inelastic mean free path (IMFP) for the photoelectrons was below 0.9 nm for all the spectra collected. For each condition, samples were equilibrated for at least 30 mins before the measurement. By taking spectra at different sample spots and comparing spectra before and after beam illumination for 2 h, we found beam damage on the sample is negligible during the measurements.

## Supplementary information

Supplementary Information

Peer Review File

## Data Availability

The data that support the findings of this study are available from the corresponding authors upon request.

## References

[1] Qiao J, Liu Y, Hong F, Zhang J (2014). A review of catalysts for the electroreduction of carbon dioxide to produce low-carbon fuels. Chem. Soc. Rev..

[2] Spichiger-Ulmann M, Augustynski J (1985). Electrochemical reduction of bicarbonate ions at a bright palladium cathode. J. Chem. Soc. Faraday Trans..

[3] Appel AM (2013). Frontiers, opportunities, and challenges in biochemical and chemical catalysis of CO_2_ fixation. Chem. Rev..

[4] Chu S, Majumdar A (2012). Opportunities and challenges for a sustainable energy future. Nature.

[5] Mistry H (2016). Highly selective plasma-activated copper catalysts for carbon dioxide reduction to ethylene. Nat. Commun..

[6] Mistry H, Varela AS, Kühl S, Strasser P, Cuenya BR (2016). Nanostructured electrocatalysts with tunable activity and selectivity. Nat. Rev. Mater..

[7] Gao S (2017). Atomic layer confined vacancies for atomic-level insights into carbon dioxide electroreduction. Nat. Commun..

[8] Yang HB (2018). Atomically dispersed Ni(i) as the active site for electrochemical CO_2_ reduction. Nat. Energy.

[9] Gao S (2016). Partially oxidized atomic cobalt layers for carbon dioxide electroreduction to liquid fuel. Nature.

[10] Liu M (2016). Enhanced electrocatalytic CO_2_ reduction via field-induced reagent concentration. Nature.

[11] Zhuang TT (2018). Steering post-C–C coupling selectivity enables high efficiency electroreduction of carbon dioxide to multi-carbon alcohols. Nat. Catal..

[12] Lieber CM, Lewis NS (1984). Catalytic reduction of carbon dioxide at carbon electrodes modified with cobalt phthalocyanine. J. Am. Chem. Soc..

[13] Hara K, Kudo A, Sakata T (1995). Electrochemical reduction of carbon dioxide under high pressure on various electrodes in an aqueous electrolyte. J. Electroanal. Chem..

[14] Hoshi N, Kato M, Hori Y (1997). Electrochemical reduction of CO_2_ on single crystal electrodes of silver Ag(111), Ag(100) and Ag(110). J. Electroanal. Chem..

[15] Back S, Yeom MS, Jung Y (2018). Understanding the effects of Au morphology on CO_2_ electrocatalysis. J. Phys. Chem. C.

[16] Hemma M (2017). Enhanced carbon dioxide electroreduction to carbon monoxide over defect-rich plasma-activated silver catalysts. Angew. Chem. Int. Ed..

[17] Favaro M (2017). Subsurface oxide plays a critical role in CO_2_ activation by Cu(111) surfaces to form chemisorbed CO_2_, the first step in reduction of CO_2_. Proc. Natl Acad. Sci. USA.

[18] Xiao H, Goddard WA, Cheng T, Liu Y (2017). Cu metal embedded in oxidized matrix catalyst to promote CO_2_ activation and CO dimerization for electrochemical reduction of CO_2_. Proc. Natl Acad. Sci. USA.

[19] Li WX, Stampfl C, Scheffler M (2003). Subsurface oxygen and surface oxide formation at Ag(111): A density-functional theory investigation. Phys. Rev. B.

[20] Bao X, Lehmpfuhl G, Weinberg G, Schlögl R, Ertl G (1992). Variation of the morphology of silver surfaces by thermal and catalytic etching. J. Chem. Soc. Faraday Trans..

[21] Schmid M (2006). Structure of Ag (111) - p(4×4): No silver oxide. Phys. Rev. Lett..

[22] Schnadt J (2006). Revisiting the structure of the p(4×4) surface oxide on Ag(111). Phys. Rev. Lett..

[23] Soon A, Todorova M, Delley B, Stampfl C (2006). Oxygen adsorption and stability of surface oxides on Cu(111): a first-principles investigation. Phys. Rev. B.

[24] Li WX, Stampfl C, Scheffler M (2002). Oxygen adsorption on Ag(111): a density-functional theory investigation. Phys. Rev. B.

[25] Andryushechkin BV, Shevlyuga VM, Pavlova TV, Zhidomirov GM, Eltsov KN (2016). Adsorption of O_2_ on Ag(111): evidence of local oxide formation. Phys. Rev. Lett..

[26] Felter TE (1982). An XPS and UPS study of the kinetics of carbon monoxide oxidation over Ag(111). Surf. Sci..

[27] Barteau MA, Madix RJ (1983). Photoelectron spectra of adsorbed carbonates. J. Electron Spectros. Relat. Phenom..

[28] Bowker M, Barteau MA, Madix RJ (1980). Oxygen induced adsorption and reaction of H_2_, H_2_O, CO and CO_2_ on single crystal Ag(110). Surf. Sci..

[29] Heine C, Lechner BAJ, Bluhm H, Salmeron M (2016). Recycling of CO_2_: probing the chemical state of the Ni(111) surface during the methanation reaction with ambient-pressure X-ray photoelectron spectroscopy. J. Am. Chem. Soc..

[30] Zhang L (2012). Electronic structure and chemical bonding of a graphene oxide-sulfur nanocomposite for use in superior performance lithium-sulfur cells. Phys. Chem. Chem. Phys..

[31] Deng X (2008). Surface chemistry of Cu in the presence of CO_2_ and H_2_O. Langmuir.

[32] Taifan W, Boily JF, Baltrusaitis J (2016). Surface chemistry of carbon dioxide revisited. Surf. Sci. Rep..

[33] Tang W, Sanville E, Henkelman G (2009). A grid-based Bader analysis algorithm without lattice bias. J. Phys. Condens. Matter.

[34] Sanville E, Kenny SD, Smith R, Henkelman G (2007). Improved grid-based algorithm for Bader charge allocation. J. Comput. Chem..

[35] Henkelman G, Arnaldsson A, Jónsson H (2006). A fast and robust algorithm for Bader decomposition of charge density. Comput. Mater. Sci..

[36] Kong D (2011). Growth, structure, and stability of Ag on CeO_2_(111): synchrotron radiation photoemission studies. J. Phys. Chem. C.

[37] Cheng T, Xiao H, Goddard WA (2016). Reaction mechanisms for the electrochemical reduction of CO_2_ to CO and formate on the Cu(100) surface at 298 K from quantum mechanics free energy calculations with explicit water. J. Am. Chem. Soc..

[38] Singh MR, Goodpaster JD, Weber AZ, Head-Gordon M, Bell AT (2017). Mechanistic insights into electrochemical reduction of CO_2_ over Ag using density functional theory and transport models. Proc. Natl Acad. Sci. USA.

[39] Ma M, Liu K, Shen J, Kas R, Smith WA (2018). In Situ fabrication and reactivation of highly selective and stable Ag catalysts for electrochemical CO_2_ conversion. ACS Energy Lett..

[40] Rosen J (2015). Mechanistic insights into the electrochemical reduction of CO_2_ to CO on nanostructured Ag surfaces. ACS Catal..

[41] Hatsukade T, Kuhl KP, Cave ER, Abram DN, Jaramillo TF (2014). Insights into the electrocatalytic reduction of CO_2_ on metallic silver surfaces. Phys. Chem. Chem. Phys..

[42] Jinghua W, Yang H, Wen Y, Yanguang L (2017). CO_2_ reduction: from the electrochemical to photochemical approach. Adv. Sci..

[43] Kresse G, Furthmüller J (1996). Efficient iterative schemes for ab initio total-energy calculations using a plane-wave basis set. Phys. Rev. B.

[44] Johnson ER, Becke AD (2006). A post-Hartree-Fock model of intermolecular interactions: inclusion of higher-order corrections. J. Chem. Phys..

[45] Grimme S, Antony J, Ehrlich S, Krieg H (2010). A consistent and accurate ab initio parametrization of density functional dispersion correction (DFT-D) for the 94 elements H-Pu. J. Chem. Phys..

[46] Kittel, C. *Introduction to Solid State Physics*, *8th edn*. (Wiley India Pvt. Limited, Hoboken NJ, 2004).

[47] Cheng T (2017). Mechanism and kinetics of the electrocatalytic reaction responsible for the high cost of hydrogen fuel cells. Phys. Chem. Chem. Phys..

[48] Cheng T, Xiao H, Goddard WA (2017). Full atomistic reaction mechanism with kinetics for CO reduction on Cu(100) from ab initio molecular dynamics free-energy calculations at 298 K. Proc. Natl Acad. Sci. USA.

[49] Cheng T, Xiao H, Goddard WA (2015). Free-energy barriers and reaction mechanisms for the electrochemical reduction of CO on the Cu(100) surface, including multiple layers of explicit solvent at pH 0. J. Phys. Chem. Lett..

[50] Cheng T, Xiao H, Goddard WA (2017). Nature of the active sites for CO reduction on copper nanoparticles; suggestions for optimizing performance. J. Am. Chem. Soc..

[51] Ping Y, Nielsen RJ, Goddard WA (2017). The reaction mechanism with free energy barriers at constant potentials for the oxygen evolution reaction at the IrO_2_ (110) surface. J. Am. Chem. Soc..

[52] Xiao H, Cheng T, Goddard WA, Sundararaman R (2016). Mechanistic explanation of the pH dependence and onset potentials for hydrocarbon products from electrochemical reduction of CO on Cu (111). J. Am. Chem. Soc..

[53] Xiao H, Cheng T, Goddard WA (2017). Atomistic mechanisms underlying selectivities in C1 and C2 products from electrochemical reduction of CO on Cu(111). J. Am. Chem. Soc..

[54] Perdew JP, Burke K, Ernzerhof M (1996). Generalized gradient approximation made simple. Phys. Rev. Lett..

[55] Grass ME (2010). New ambient pressure photoemission endstation at Advanced Light Source beamline 9.3.2. Rev. Sci. Instrum..

