# Supplementary material for: Dramatic differences in carbon dioxide adsorption and initial steps of reduction between silver and copper
Source: Nat Commun. 2019 Apr 23;10:1875. doi: 10.1038/s41467-019-09846-y (PMC6478877; doi:10.1038/s41467-019-09846-y)
Supplement: Supplementary file 1 — Supplementary Information [file 41467_2019_9846_MOESM1_ESM.pdf]

Supplementary Information for

“Dramatic Differences in CO_2 adsorption and initial steps of reduction between silver and copper”

Ye *et al.*

Supplementary Figures

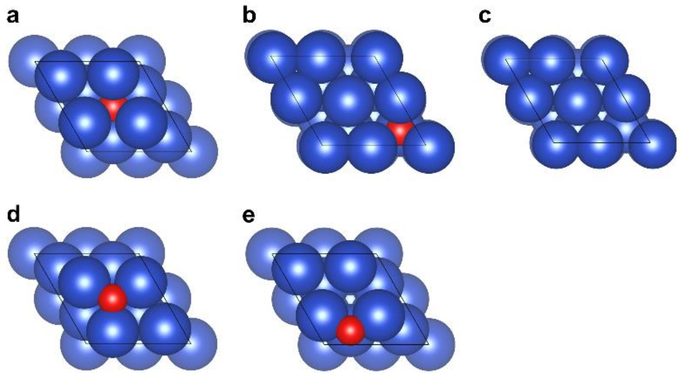

Supplementary Figure 1: The subsurface and surface O in Cu system. The configurations represent **(a)** the octahedron subsurface oxygen, **(b)** tetrahedron subsurface oxygen (O below 3-fold site), **(c)** tetrahedron subsurface oxygen (O below top site), **(d)** fcc surface oxygen, and **(e)** hcp surface oxygen, respectively. The stability of these species are summarized in the **Supplementary Table 1**.

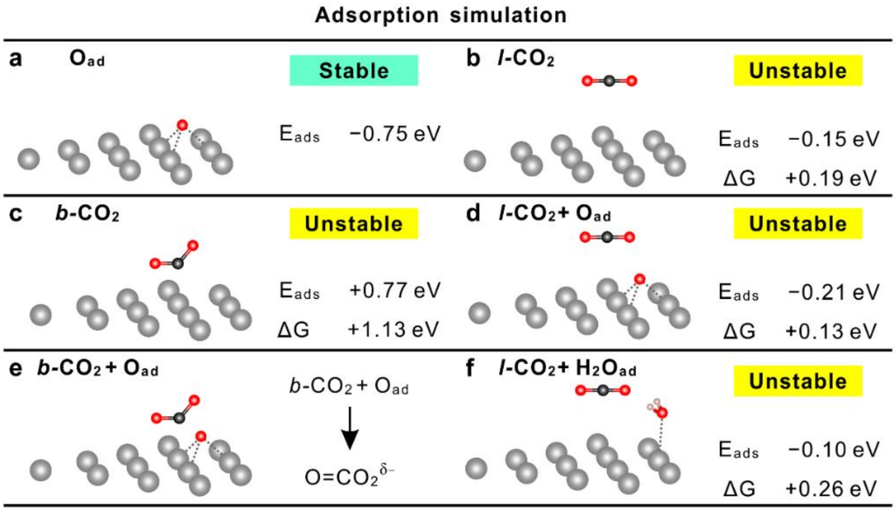

Supplementary Figure 2. The QM predictions of adsorbates on the Ag surface. a, Surface O adsorbed (O_{ad}) on Ag surface on-top three-fold (η_3) site. **b,c,** The optimized structure for *l*- and *b*- CO_2 on pristine Ag surface. Both *l*- and *b*- CO_2 are found to be unfavorable with $E_{ads} = -0.15$ eV and $\Delta G = +0.19$ eV, and $E_{ads} = +0.77$ eV and $\Delta G = +1.13$ eV, respectively. **d,** The optimized structure of *l*-CO_2 on Ag surface in the presence of isolated surface O_{ad}. This configuration is found to be unfavorable with $\Delta E_{ads} = -0.21$ eV, but $\Delta G = +0.13$ eV. Thus a pressure of ~30 Torr would be required to stabilize *l*-CO_2 on the O/Ag surface at 298 K. **e,** The optimized structure of *b*-CO_2 on Ag surface in the presence of isolated surface O_{ad}. *b*-CO_2 interact with the on-top surface O_{ad} atoms to form a chemisorbed surface carbonic acid-like $O{=}CO_2^{\delta-}$ species as shown in main text **Figure 1c and 2a**. **f.** The optimized structure of *l*-CO_2 on Ag surface in the presence of surface H_2O. We find that (*l*-CO_2)-(H_2O) remains unstable on Ag surface ($E_{ads} = -0.10$ eV and $\Delta G = +0.26$ eV), even with the help of H_2O. The hydrogen, carbon, oxygen, and silver atoms were represented with rosy brown, black, red, and gray balls, respectively.

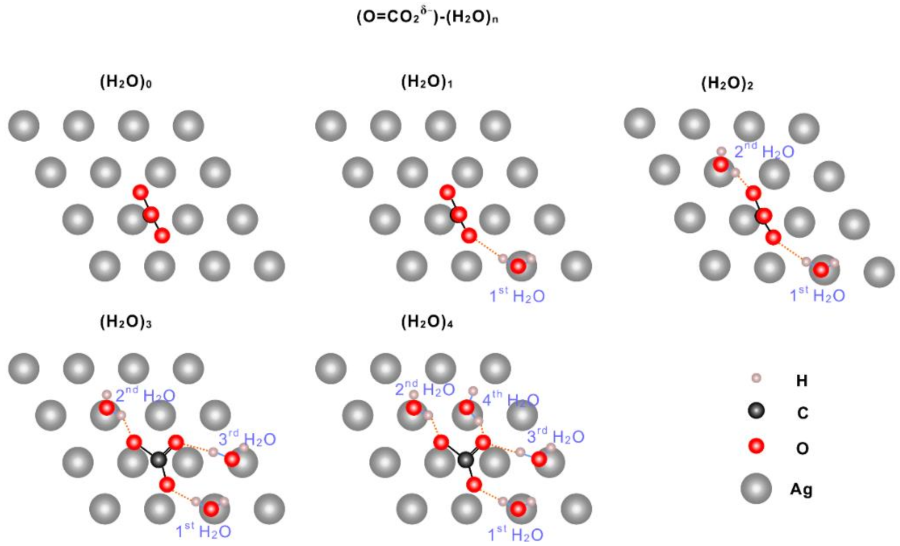

Supplementary Figure 3. The configuration of $O{=}CO_2^{\delta-}$ stabilizing 0-4 water molecules illustrated in top view. $O{=}CO_2^{\delta-}$ is a carbonic acid-like structure with a $C{=}O_{up}$ double bond pointing up while the other two O bind to adjacent three fold Ag (111) sites. Adding the 1st and 2nd don't change the $O{=}CO_2^{\delta-}$ structure but forming two hydrogen bonds (shown as orange dashed lines) with each O bonded to the Ag surface. Adding 3rd and 4th H_2O force the C=O bond to rotate from being perpendicular to the surface to being tilted nearly parallel to the surface, allowing the formation of HB from a 3rd and 4th surface H_2O to the two sp^2 lone pairs on the C=O unit. The hydrogen, carbon, oxygen, and silver atoms are represented with rosy brown, black, red, and gray balls, respectively. The C-O (and C=O), O-H, and hydrogen bonds are represented with black, blue, and orange sticks, respectively.

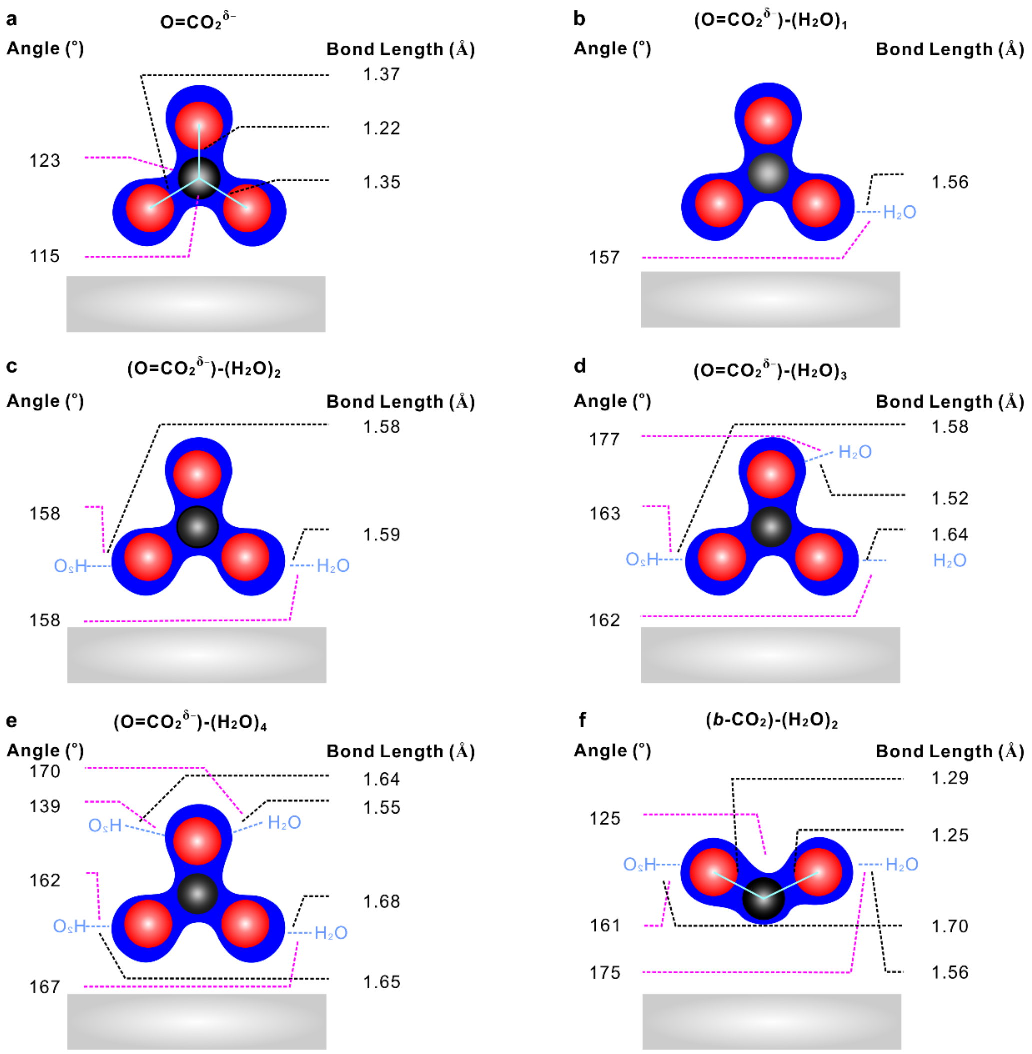

Supplementary Figure 4. The geometrical structure of various adsorbates on the Ag surface. The bond angel and bond length for various adsorbates on Ag are illustrated.

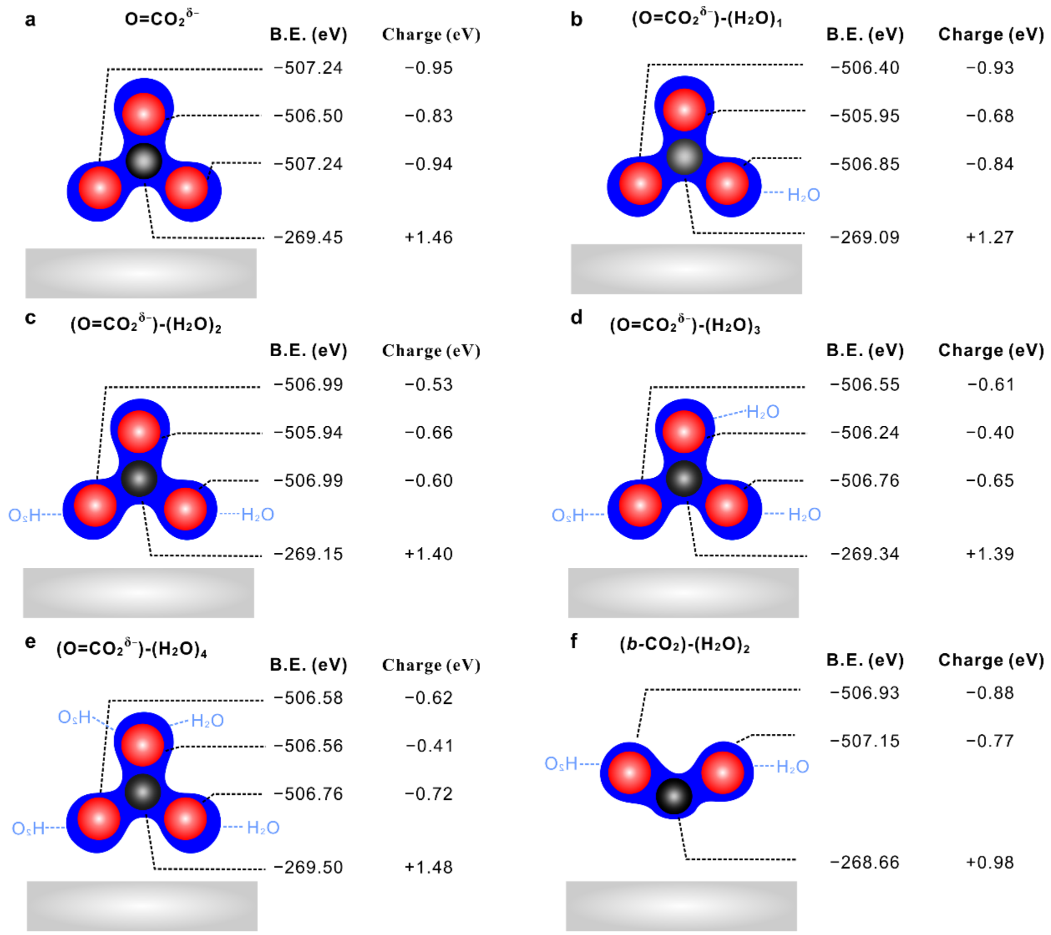

Supplementary Figure 5. The electronic structure of various adsorbates on Ag surface. The charges on the C and O in the various adsorbates on Ag are illustrated, the corresponding simulated BEs are displayed as well.

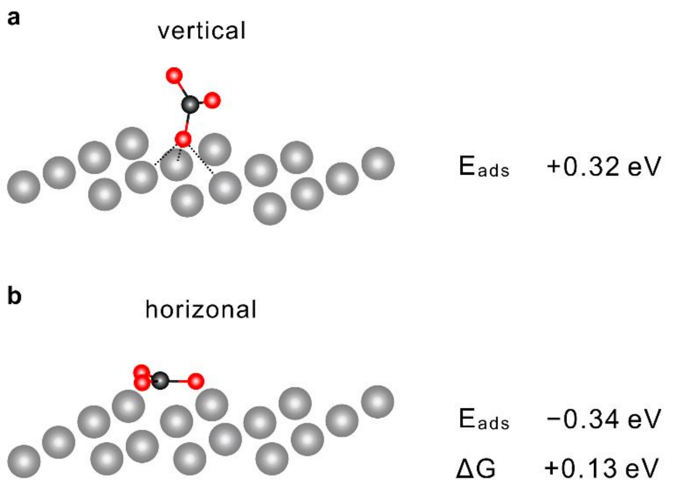

Supplementary Figure 6. The QM predictions of CO_3 configurations on the Ag surface. a, vertical CO_3 configuration on Ag surface with one O on the surface. **b,** The horizontal CO_3 configuration on Ag surface with three C-O bonds parallel to the surface. Both configurations are not stable under the condition of 0.3 Torr CO_2 at 298K.

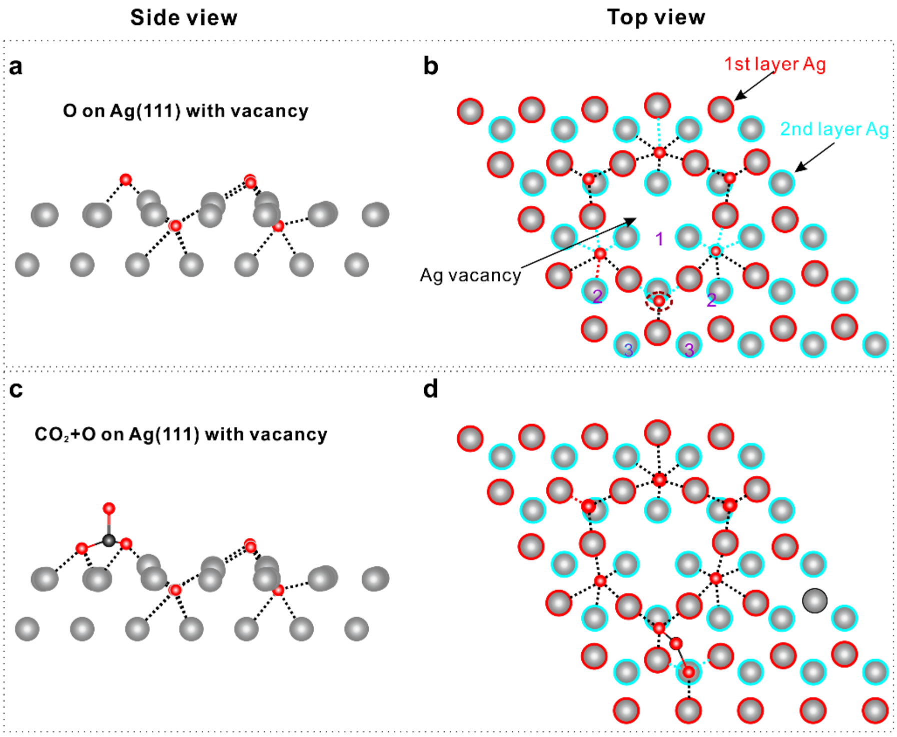

Supplementary Figure 7. The Ag vacancy in a 7×7 Ag (111) unit cell. (a) Side view and **(b)** top view of Ag (111) surface with 6 oxygen surrounding one Ag vacancy. **(c)** Side view and **(d)** top view of a starting CO_3 configuration on Ag (111) surface with 6 oxygen surrounding one Ag vacancy. The adsorption of CO_2 on the Ag (111) surface with an Ag vacancy induced by oxygen adsorption is found to be unstable on this structure.

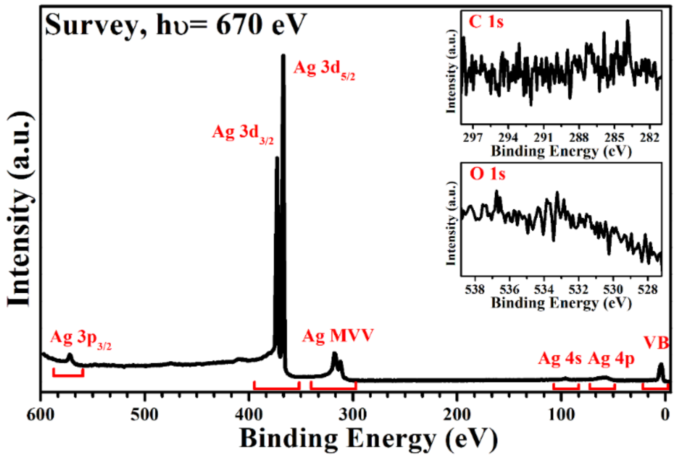

Supplementary Figure 8: The survey and high resolution C 1s and O 1s scans of the pristine Ag foil. The Ag foil is cleaned prior to each experiment by repeated Ar sputtering and vacuum annealing. The Ag surface is characterized by XPS to ensure no detectable contamination on the surface. The survey with a binding energy range of −10 to 600 eV, and high resolution scans of C 1s and O 1s are recorded at photon energy of 670 eV. The energy scale of the spectra is calibrated using the Ag 3d $_{5/2}$ peak locating at 368.2 eV. The survey spectra show only Ag signals, including core level peaks and an auger peak. No detectable C- and O- based contamination are observed in the high resolution scans recorded in the insets.

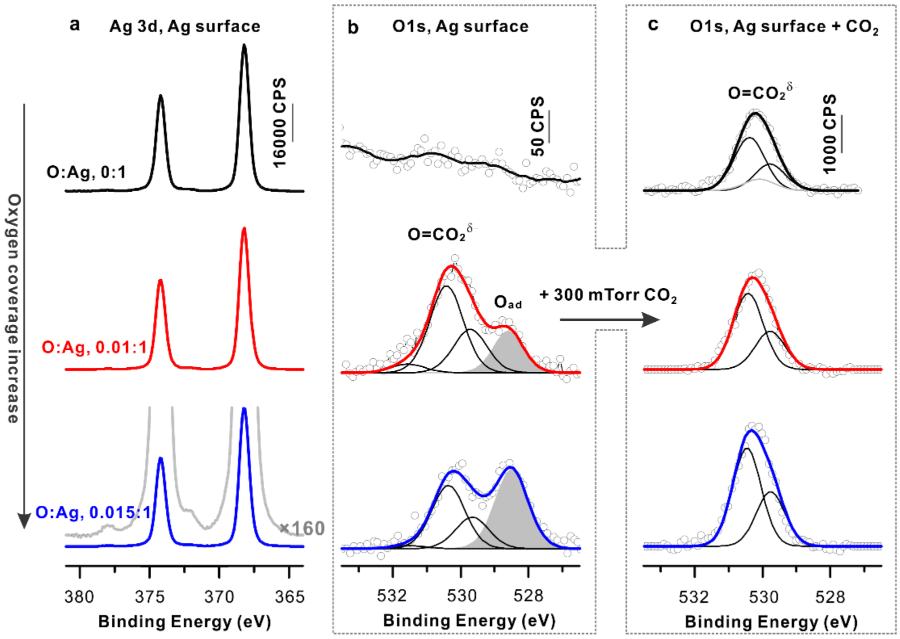

Supplementary Figure 9. APXPS of pristine and oxygen-covered Ag surfaces and the adsorbates on them. a, Ag 3d spectra of pristine and oxygen treated Ag surface. The region of the loss feature peaks is enlarged to indicate the metallic feature of the Ag surface after O_2 treatment. **b,** O1s spectra of pristine and oxygen treated Ag surface. The spectra show signals of atomically adsorbed O on the surface, and two peaks from $O{=}CO_2^{\delta-}$, respectively. **c,** O1s spectra of pristine and oxygen treated Ag surface after CO_2 adsorption. The O_{ads} peak showing up before the CO_2 adsorption disappears after CO_2 adsorption. The signal attenuation due to0.3 Torr CO_2 in the chamber has been calibrated by applying the substrate signal decay.

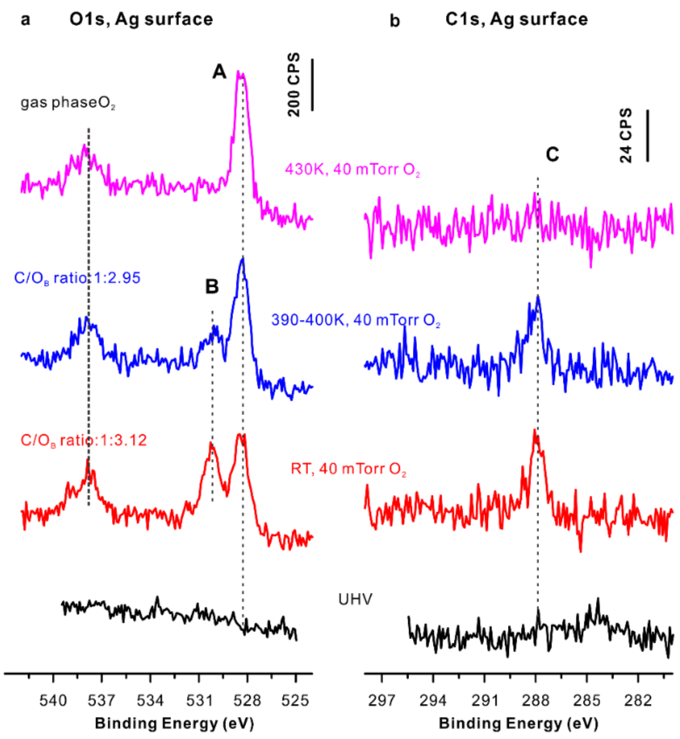

Supplementary Figure 10. APXPS of Ag surfaces during O_2 dose. a, O 1s and **b,** C 1s spectra taken at UHV, 40 mTorr O_2 at room temperature, 40 mTorr O_2 at around 400 K, and 40 mTorr O_2 at 430K are recorded as black, red, blue, and pink, respectively, from bottom to top.

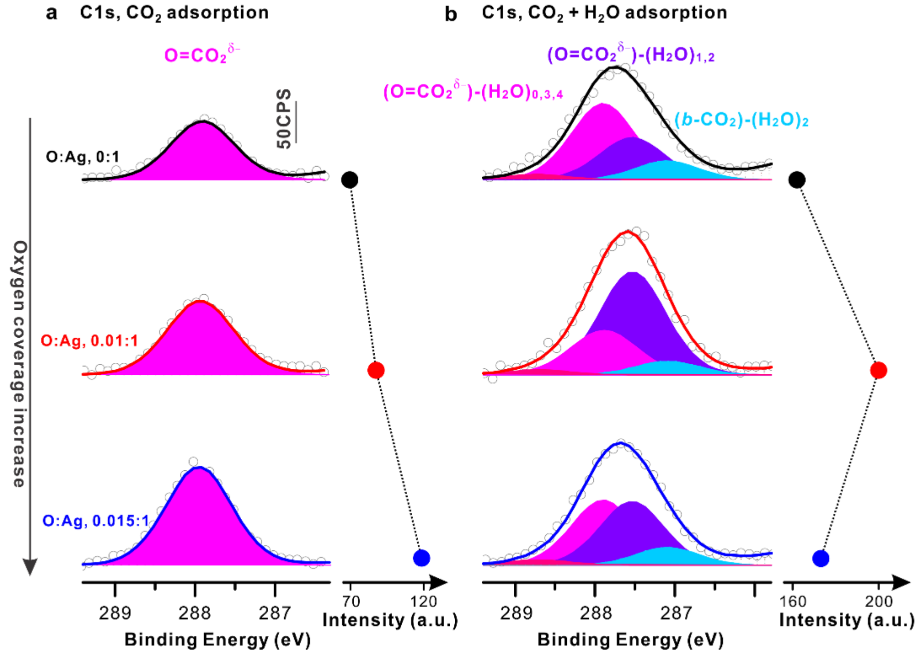

Supplementary Figure 11: C 1s spectra of adsorbates on Ag surface after CO_2 adsorption both alone and in the presence of H_2O. a, C 1s spectra of CO_2 adsorption on various Ag surfaces. As the surface O_{ad} coverage is increased, additional CO_2 adsorbed on the surface to form $O{=}CO_2^{\delta-}$ while suppressing CO_2 dissociated surface carbon formation, thus leading to an increase in $O{=}CO_2^{\delta-}$ signals. **b,** C 1s spectra of CO_2 + H_2O adsorption on various Ag surfaces. The total adsorbate signal from CO_2+H_2O co-adsorption shows a volcano-shaped dependence on the surface oxygen coverages. We explain this in terms of the competitive adsorption among the surface species. Given a coverage of O_{ad}, we expect adsorption competition between CO_2 reacting with O_{ad} to form $O{=}CO_2^{\delta-}$ requiring 2 sites, one to four H_2O coordinating to $O{=}CO_2^{\delta-}$ requiring 3 to 6 sites, and H_2O reacting with O_{ad} to form two OH_{ad} requiring 2 or 3 sites. Thus increased amounts of $O{=}CO_2^{\delta-}$ block further adsorption of H_2O_{ads}, which suppresses the effect of H_2O_{ads} in stabilizing the surface adsorbates. For the clean Ag surface, surface adsorption is suppressed by the formation of sp^2 carbon from CO_2 dissociation, showing decreased amounts of surface adsorbates compared to oxygen covered Ag surfaces. Thus, having too many surface O (more than 0.15 O per Ag) or none at all both lead to decreased adsorbates on the surface.

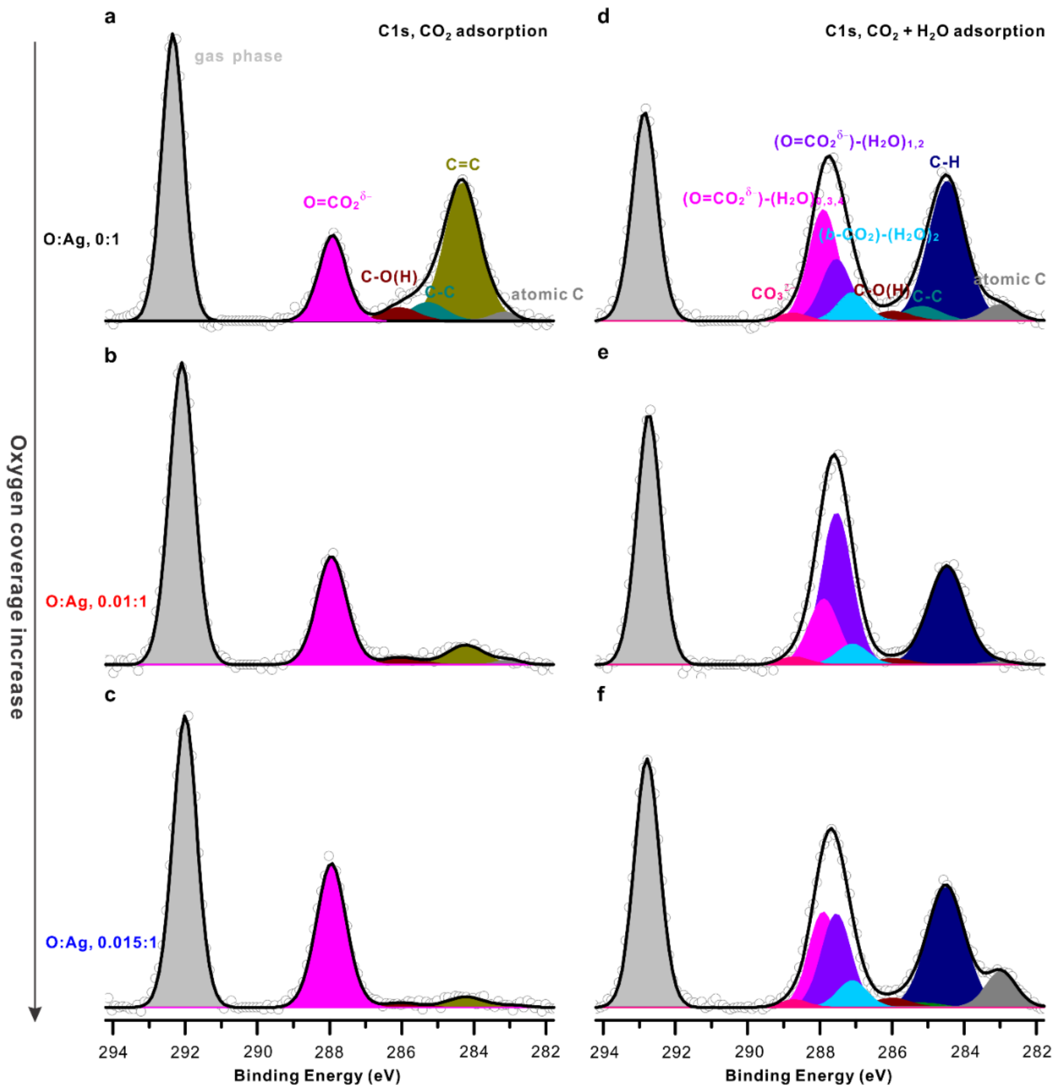

Supplementary Figure 12. The detailed peak deconvolution of the C 1s spectra recorded on various Ag surface under CO_2 adsorption alone and in the presence of H_2O. The C 1s spectra are divided into three parts: the surface reaction products, surface adsorbates, and gas phase peak. The chemical species can be assigned as atomic C (283.0 eV), sp^2 C=C (284.2 eV), sp^3 C-C (285.2 eV), C-O(H) (286.0 eV), $O{=}CO_2^{\delta-}$ (287.9 eV), *g*-CO_2 (292-293 eV)[1, 2]. During the coadsorption of CO_2 and H_2O, a new species appears at 284.5 eV between the peaks of sp^2 C=C and sp^3 C-C. Detailed assignment for this species has not been made, but it may be related to the C-H bond formation, due to the reaction between sp^2 C=C and H_2O on Ag surface. We also have observed the ionic carbonate species at 288.7 eV in the C 1s XPS spectra. The appearance of CO_3^{2-} may originate from some side reactions, which has been reported previously. The energy difference of ~0.8 eV between the CO_3^{2-} and $O{=}CO_2^{\delta-}$ peaks provides direct evidence that these two species have completely different electronic structure properties.

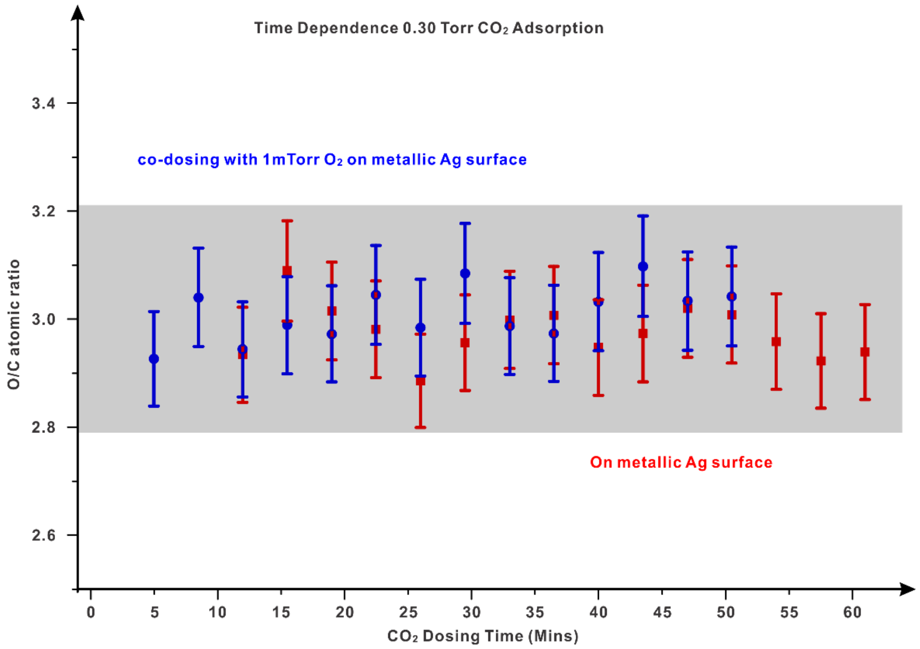

Supplementary Figure 13. The O:C atomic ratio of surface adsorbates as a function of CO_2 dosing time. The O:C atomic ratio shows around 3:1 during this process, further proving that the surface adsorbate has a configuration of CO_3.

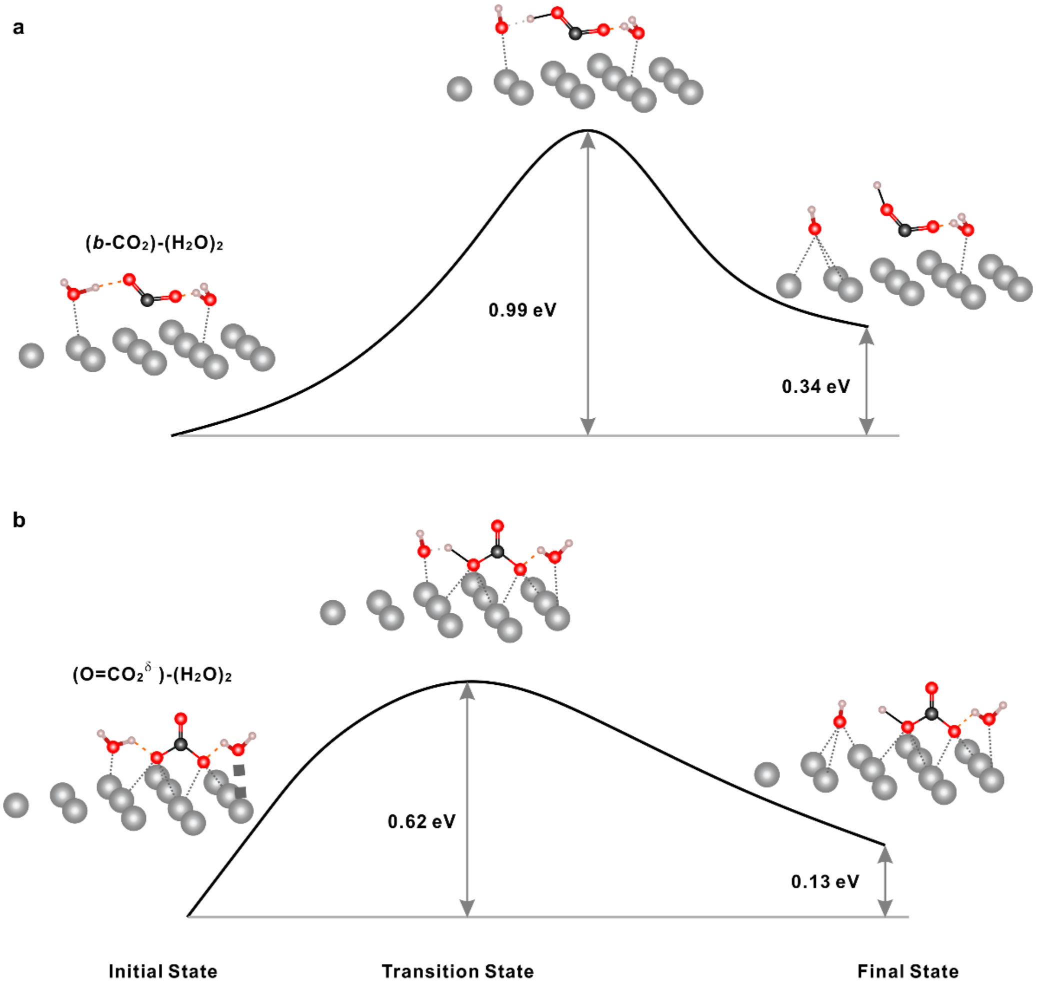

Supplementary Figure 14. Energy barrier of H transferring process originating from *b*-CO_2 and $O{=}CO_2^{\delta-}$. a, Hydrogenation process of *b*-CO_2 with one H transferred from attached H_2O molecule to form the HOCO intermediate plus OH_{ad}. The energy barrier for this process is 0.99 eV. In the initial state, the O-H bond lengthen for the attached H_2O is 1.025 Å, while the HB lengthen is 1.557 Å. In the transition state with a H transferred, the distance between the attached H and the O in H_2O is 2.856 Å. While this distance increases to 3.823 Å in the final state. **b,** Hydrogenation process of $O{=}CO_2^{\delta-}$ with one H transferred from attached H_2O molecule to form the (C=O)(O)(OH) intermediate plus OH_{ad}. The energy barrier for this process is 0.62 eV. In the initial state, the O-H bond lengthen for the attached H_2O is 1.023 Å, while the HB lengthen is 1.582 Å. In the transition state with a H transferred, the distance between the attached H and the O in H_2O is 2.576 Å. While this distance increases to 4.357 Å in the final state.

Supplementary Tables

Supplementary Table 1: DFT predicted energies for O atom at various positions on and in the Cu(111) surface. The configuration of $O_{sub, octa}$, $O_{sub, tetra, 3 fold}$, $O_{sub, tetra, top}$, $O_{surf, fcc}$, and $O_{surf, hcp}$ are displayed in the Supplementary **Figure 1 (a)-(e)**, respectively. Because DFT does not describe the O-O bond strength accurately, we define the energy of the O atom species relative to O_2 as ΔE = E(surface species and surface) − E(surface) − ½ E(O_2 molecule from experiment). The coverage of oxygen used for the simulation is 1/4 ML.

Structure	PBE		PBE-D3		Garza et.al	
	Energy (eV)	ΔE (eV)	Energy (eV)	ΔE (eV)	PBE (eV)*	(SCAN+rVV1) (eV)**
**O atom**	−1.68	N/A	−1.68	N/A	N/A	N/A
**O-O bond**	−6.50	N/A	−6.50	N/A	N/A	N/A
**O-O bond (exp)**	−5.16	N/A	−5.16	N/A	N/A	N/A
**O_2 molecule (exp)**	−8.52	N/A	−8.52	N/A	N/A	N/A
**O_2 molecule**	−9.86	N/A	−9.87	N/A	N/A	N/A
**Cu**	−55.27	N/A	−62.24	N/A	N/A	N/A
**(a) $O_{sub, octa}$**	−60.62	−1.08	−67.68	−1.18	−0.30	−4.55
**(b) $O_{sub, tetra, 3 fold}$**	−60.36	−0.83	−67.33	−0.83	0	−4.03
**(c) $O_{sub, tetra, top}$**	unstable	N/A	unstable	N/A	N/A	N/A
**(d) $O_{surf, fcc}$**	−61.85	−2.32	−69.03	−2.53	−1.84	−5.93
**(e) $O_{surf, hcp}$**	−61.72	−2.19	−68.89	−2.39	N/A	N/A
**$O_{Third layer, octa}$**	−60.01	−0.48	−66.96	−0.46	N/A	N/A

*reference to subsurface O at tetrahedron site (O below 3-fold site)

**reference to atomic O

Supplementary Table 2. Vibrational frequency for all possible species on the surface with CO_2 adsorption both alone and in the presence of H_2O.

**CO_2 adsorption**		
**Species**	**Bond**	**vibrational frequency (cm^{-1})**
$O{=}CO_2^{\delta-}$	C-O stretch	908.06
$O{=}CO_2^{\delta-}$	C=O stretch	1663.51
**CO_2+H_2O adsorption**		
**Species**	**Bond**	**vibrational frequency (cm^{-1})**
H_2O	O-H stretch	3709.04 (anti); 3612.91 (sym)
	H-O-H bend	1555.86
$O{=}CO_2^{\delta-}+H_2O$	C-O stretch	900.79
	C=O stretch	1659.96
	O-H stretch	3777.14; 2730.96
$O{=}CO_2^{\delta-}+2H_2O$	C-O stretch	992.578159
	C=O stretch	1669.03
	O-H stretch	3772.44, 3767.26
$O{=}CO_2^{\delta-}+3H_2O$	C-O stretch	931.07
	C=O stretch	1610.86
	O-H stretch	3765.34; 2743.9; 3764.92
$O{=}CO_2^{\delta-}+4H_2O$	C-O stretch	935.66
	C=O stretch	1600.43
	O-H stretch	3762.63; 2765.34; 3720.03
b-CO_2+2H_2O	O-H stretch	3763.64; 3761.32; 3180.168; 2739.99

Supplementary Table 3. The summary of the stability and free energy of possible surface adsorbates on Ag and Cu surfaces.

	Ag		Cu	
**adsorbate**	**Stability**	**ΔG (eV)**	**Stability**	**ΔG (eV)**
***l*-CO_2**	Not stable	+0.19 (PBE-D3)	Stable with sublayer oxygen	−0.39 (M06L)
***b*-CO_2**	Stable with two hydrogen bonds	−0.18 (PBE-D3)	Stable with hydrogen bond and sublayer oxygen	−0.06 (M06L)
**$O{=}CO_2^{\delta-}$**	Stable with surface O	−0.28 (PBE-D3)	Not stable	+1.33 (PBE-D3)
	Stable with 1 hydrogen bonds	−0.43 (PBE-D3)		
	Stable with 2 hydrogen bonds	−0.48 (PBE-D3)		
	Stable with 3 hydrogen bonds	−0.37 (PBE-D3)		
	Stable with 4 hydrogen bonds	−0.19 (PBE-D3)		

Supplementary Notes

Supplementary Note 1.
Stabilities of surface and subsurface O in Cu and Ag.

The stability of subsurface oxygen in Cu was questioned recently in study performed by Garza et al.[3]. This may have caused confusion in the community, so we want to clarify the Cu results for O atoms on Cu surfaces in a vacuum, comparing the differences and consistencies between our previous works with Garza's.

We are interested in the existence of both subsurface and surface oxygen on Cu surface. Our previous QM calculations on Cu used the advanced M06 version of DFT theory optimized to describe both van der Waals attraction and reaction pathways, whereas Garza et al. used the PBE method for oxygen and the SCAN+rVV10 functional for physisorption of CO_2 on copper. Our previous QM calculations were carried at experimental conditions with gas phase CO_2 and H_2O (total pressure 0.7 Torr, and room temperature), which can be compared directly to the results in this this current manuscript. On the other hand, Garza et.al carried out the calculations with electrolyte and external potential, which, although valuable, is not directly comparable.

Our work calculated the free energy of binding of the various species showing the stability of the various species under the experimental pressures and temperatures. This led to excellent agreement with the APXPS chemical shifts. The experimental evidence of subsurface oxide is quite clear from the O 1s spectra characterizations. Also, the experimental results of adding additional O experimentally confirmed our QM predictions. Indeed, Garza's work and our results for O interacting with Cu surfaces in a vacuum are quite consistent, as shown in Supplementary **Table 1**. We both found that the *b*-CO_2 can only be stable with extra charge transferred to bend the CO_2 molecular structure. While the M06 DFT finds that subsurface O changes the Cu valence state to provide extra charge, Garza applied an external potential that provides extra charge to stabilize the bent configuration. Although our Cu experimental data does not include electrolyte and applied potential, our experiment together with the theory does show that extra charge can stabilize the *b*-CO_2 with H_2O. Summarizing. The previous experiments prove the existence of subsurface O for Cu surfaces in a vacuum that the M06 DFT also finds and the QM and APXPS are fully consistent.

For the Cu(111) and Ag(111) surfaces, we examined the stability of surface and subsurface O. For Ag, subsurface O is not stable and transfers to form surface O without an energy barrier. For Cu(111) the DFT predicted energies for O atom at various positions on and in the Cu(111) surface (Supplementary **Figure 1**) are summarized in Supplementary **Table 1**. O atom on the surface is bound by 2.53 eV with respect to ½ O_2 (gas phase) while subsurface O is bound by 0.83 eV in the tetrahedral site and by 1.18 eV in the octahedral site. These results are in line with the results by Garza et al..[3] Thus, both studies reach the same conclusion that formation of subsurface O on Cu is strongly favored thermodynamically compared to gas phase O_2, but subsurface O is less stable than surface O. The appearance of subsurface O in the Cu is also further evidenced by many experimental studies.[4-7]

Supplementary Note 2.
Stabilities and properties of adsorbates on Ag.

In the case of O on Ag surface. We find that sublayer O (which stabilized both the *l*- and *b*- CO_2 in Cu system) is not stable on Ag, quantum mechanics (QM) finds that putting an O in a Ag sublayer site goes without a barrier to an on-top three-fold (η_3) site (Ag-O = 2.14 Å) with $\Delta E = -1.46$ eV. Previous studies included some discussion on the subsurface O in the Ag system, which is introduced by through the grain boundary, defects in the structure, and diffusion of the surface oxygen into the bulk. These cases required moderate to high temperature and high oxygen coverage[8, 9]. Moreover, Li et al. performed a series of studies examining the stability of subsurface oxygen in Ag and found that the transition barrier from surface oxygen to subsurface oxygen on Ag(111) surface is +0.86eV, whereas the reverse barrier from subsurface to surface oxygen is only +0.18eV, leading to the population of surface oxygen is around $\exp(34)=5.8\times10^{14}$, which is around 10^{14} times more than subsurface oxygen[10]. It is found that crystal expansion is needed to stable subsurface oxygen, where the high oxygen coverage is needed[11, 12].

In the case of *l*-CO_2 on Ag surface. The optimized structure for physisorbed CO_2 on the clean surface has an O-C-O angle of 180° with 1.177Å CO bonds, essentially the same for the PBE-D3 calculation on gas phase CO_2 (1.176Å). This linear CO_2, denoted *l*-CO_2, is physisorbed parallel to the Ag surface, at a height of 3.08Å above Ag surface (Supplementary **Figure 2b**). The calculated QM adsorption energy is $\Delta E_{ads} = -0.15$ eV. Including the phonon corrections for zero-point energy (ZPE) and pressure leads to a free energy of $\Delta G_{298} = +0.19$ eV, so this state is not observed in our experiments. The core levels are C1s = 270.82 eV and O1s = 509.70 eV. These energetics would require a CO_2 pressure of ~500 Torr for the *l*-CO_2 be stabilized on the clean Ag surface at 298K. This agrees with previous reports that at UHV condition *l*-CO_2 was only observed at temperatures below 130K.

In the case of *l*-CO_2 on Ag surface with surface oxygen. In the presence of isolated surface O, we found that *l*-CO_2 has $\Delta E_{ads} = -0.21$ eV with C1s = −270.26 eV, but $\Delta G = +0.13$ eV. Thus a pressure of ~30 Torr would be required to stabilize *l*-CO_2 on the O/Ag surface at 298K. This contrasts with observations for Cu, where sublayer O stabilized the adsorption of *l*-CO_2 on Cu surface under 0.7 Torr CO_2 partial pressure at 298 K. This attraction resulted from the subsurface O in a tetrahedral site inducing Cu^+ character into the single Cu above it on the surface, which stabilized the *l*-CO_2. This oxygen promotion effect was not observed in this work because the O is chemisorbed on top of the Ag, which does not change the valence state of Ag.

In the case of *b*-CO_2 on Ag surface. We also investigated the stability of the *b*-CO_2 on Ag surface. The optimized structure for *b*-CO_2 is 2.012Å above Ag surface for C atom, and 2.145Å and 2.945Å for the two O atoms, respectively (Supplementary **Figure 2c)**. The predicted C-O bond lengths are 1.283Å and 1.245Å, respectively, significantly increased compared to those of *g*- and *l*-CO_2 (1.176Å). The DFT finds that *b*-CO_2 is unfavorable by $E_{ads} = +0.77$ eV. Thus *b*-CO_2 is not stable the on pristine Ag surface.

In the case of CO_3 configurations on Ag surface. We carried out several QM calculations for the configuration of CO_3 structure on Ag surface from CO_2 adsorption to a chemisorbed O atom. We found that the only stable CO_3 structure is with two O on the surface and one C=O bond perpendicular to the surface as shown in **Figure 2**. We found that positioning one O on the surface and two C-O bonds pointing to vacuum is not stable with an adsorption energy of +0.32 eV (Supplementary **Figure 6**). Minimizing this monodentate structure leads to the bidentate structure. We also carried out QM calculations for the horizontal configuration with three C-O bonds parallel to the surface. This configuration is also not stable with an adsorption energy of −0.34 eV but $\Delta G = +0.13$ eV. Minimizing this structure leads to the bidentate structure.

In the case of CO_2 adsorption on Ag surface with Ag vacancy. Some experimental studies have reported that O adsorption on Ag (111) surface induces the formation of Ag vacancies, which may act as active sites for CO_2 adsorption. We investigated the interaction of gas phase CO_2 with this oxygen-covered defective Ag surface. We examined all possible binding sites for forming the CO_3 structure. Prior to CO_2 adsorption we obtained a structure similar to that reported in the previous work, [13] where 6 surface oxygen atoms surrounding each Ag vacancy (Supplementary **Figure 7**). In the top view configuration, the first and second layer Ag atoms are highlighted by red and cyan outlines, respectively. As well established in the discussion above, the only stable configuration for CO_2 adsorption on Ag surface is the CO_3 structure having 2 oxygens bound to the three-fold site and 1 oxygen standing straight up and double bonded to the carbon. Thus, as labeled in Supplementary **Figure 7**, three sites around each O are available for CO_2 attachment. Position 1 and 2 are found not to be possible for placing a CO_3 because of spatial constraints. From the side view, it is clear that the vacancy structure has Ag popping out in the Z direction, which creates a distortion that collides with the position of the C atom, making binding of CO_3 impossible for position 1 or 2. We attempted to put CO_2 at position 3 to form CO_3 structure as an initial structure (Supplementary **Figure 7**). This structure is not stable and relaxed to a *l*-CO_2 and a surface O, with ΔG = +0.44 eV. We conclude that Ag vacancy surrounded with 6 oxygen atoms cannot act as an active site for CO_2 adsorption.

Supplementary Note 3.
Geometrical and electronical structures of various adsorbates on Ag.

The geometrical and electronical structures of various adsorbates on Ag are shown in the Supplementary **Figure 4 and 5**, respectively. Specifically, they are shown as followings.

In the case of $O{=}CO_2^{\delta-}$ on Ag. The $O{=}CO_2^{\delta-}$ has a $C{=}O_{up}$ double bond (1.222 Å) pointing up while the other two O bind to adjacent three fold Ag sites with C-O lengths of 1.365 Å and 1.354 Å, respectively, and O-Ag distances of 2.276 Å. The two O_{down} have charges of −0.95 e^- and −0.94 e^-, respectively, while O_{up} has a charge of −0.83 e^-. The corresponding O1s core level: two O1s= −507.24 eV, and one O1s= −506.50 eV.

In the case of $(O{=}CO_2^{\delta-})$-$(H_2O)_1$ on Ag. The hydrogen bond between H_2O_{ads} and $O{=}CO_2^{\delta-}$ did not change the structure of $O{=}CO_2^{\delta-}$. The hydrogen bond showed H-O bond length of 1.561Å, with an O-H-O angle of 157.5°. The charge on the O_{down} (O_1) hydrogen bonded to H_2O_{ads} (O_4) changes from 0.95e^- to 0.84e^- leading to O1s(1) = −506.85 eV, while the oxygen in the C=O bond (O_3) changes from 0.83e^- to 0.68e^- with O1s(3) = −505.95 eV, and the charge on the other O_{down} (O_2) remains unchanged with O1s(2) = −506.40 eV. The H_2O_{ads} (O_4) leads to O1s(4) = −507.29 eV.

In the case of $(O{=}CO_2^{\delta-})$-$(H_2O)_2$ on Ag. Forming another hydrogen bond did not change the structure of $O{=}CO_2^{\delta-}$ as well. The hydrogen bonds showed H-O bond length of 1.585 Å and 1.583 Å with O-H-O angles of 157.5° and 158.3°, respectively. The O1s of the C=O has charge of −0.53e^-, with the O1s = −505.94 eV, while the two O attaching to H_2O have charge of −0.6e^- and −0.66e^-, respectively, with two O1s = −506.99 eV, and the two O of H_2O have O1s = −507.43 eV.

In the case of $(O{=}CO_2^{\delta-})$-$(H_2O)_3$ on Ag. Adding a 3rd H_2O bends the terminal O=C toward to Ag surface to form a hydrogen bond. The hydrogen bonds showed H-O bond length of 1.576 Å and 1.637 Å with O-H-O angles of 163.3° and 161.8° for first two added waters, and H-O bond length of 1.517 Å with O-H-O angles of 176.8° for third added water, respectively. Besides, the third water formed another hydrogen bond with adjacent H_2O showed H-O bond length of 1.970 Å and O-H-O angles of 140.9°. The O1s of the C=O has charge of −0.40e^-, with the O1s = −506.24 eV, while another two O have charge of −0.61e^- and −0.65e^-, respectively, with two O1s = −506.55 eV and −506.76 eV, and the three O of H_2O have O1s = −507.08 eV, −507.25 eV and −507.32 eV, respectively.

In the case of $(O{=}CO_2^{\delta-})$-$(H_2O)_4$ on Ag. Forming another hydrogen bond with fourth H_2O did not change the structure of $O{=}CO_2^{\delta-}$. The hydrogen bonds showed H-O bond length of 1.646 Å and 1.678 Å with O-H-O angles of 162.1° and 166.9° for first two added waters, and H-O bond length of 1.547 Å and 1.638 Å with O-H-O angles of 170.4° and 139.4° for two additional waters, respectively. The O1s of the C=O has charge of −0.41e^-, with the O1s = −506.56 eV, while another two O have charge of −0.62e^- and −0.72e^-, respectively, with two O1s = −506.58 eV and −506.76 eV, and the four O of H_2O have O1s = −507.06 eV, −507.14 eV, −507.57 eV and −507.42 eV, respectively.

In the case of (b-CO_2)-$(H_2O)_2$ on Ag. This configuration has C-O bond length of 1.293Å and 1.245Å and an O-C-O angle of 125.1º. The two hydrogen bond lengths are 1.704Å, and 1.556Å, with O-H-O angles of 160.7º and 174.7º respectively. Two O of the b-CO_2 have charges of $0.88e^-$ and $0.77e^-$, respectively, with O1s(1) = −506.93eV , O1s(2) = −507.15 eV, the O of the corresponding H_2O_{ads} have O1s(3) = −507.48 eV, O1s(4) = −507.81 eV.

Supplementary Note 4.
Oxygen species on the oxygen-covered Ag surfaces.

O 1s spectra recorded on oxygen covered Ag surface showed three peaks locating at 528.5 eV, 530.3 eV, and 531.5 eV, respectively. The peak locating at 528.5 eV is between the previous observed signal of Ag_2O and signal from Ag (111)- p(4 × 4)- O surface reconstruction[14]. However, we haven't observed any changes on the Ag peak (as shown in Supplementary **Figure 9a**), which may show a peak lower than the bulk metallic peak if these two cases appeared. Thus, we tentatively assign this peak as the atomically adsorbed O on the surface[15-17]. The peak locating at 530.3 eV was assigned to the peak of $O{=}CO_2^{\delta-}$, which has also been reported previously[17]. This assignment is supported by checking the C 1s signal and the C:O atomic ratios, which are around 1:3 during the O_2 adsorption process (Supplementary **Figure 10**). Since the peak position of this species in both the C 1s and O 1s spectra is located at the identical position as those we observed later with CO_2 adsorption, we are confident to assign them to $O{=}CO_2^{\delta-}$. This is further evidenced by its unstable of peak B above 430K. This is against the previous assigned bulk dissolved O peak, locating at similar position, is stable at up to 800K[8, 9]. By applying the sensitivity factors for both Ag 3d and O 1s, which are about 1.8 and 0.32, respectively, under photon energy of 670 eV, the Ag:O atomic ratio is around 0.01 and 0.015 for low and high oxygen covered surface, respectively. The maintenance of the metallic state of Ag and the low coverage of oxygen on the Ag further ruled out the formation of the Ag (111)-p(4×4)-O surface reconstruction.

Supplementary References:

1. Taifan, W., Boily, J.-F. & Baltrusaitis, J. Surface chemistry of carbon dioxide revisited. *Surf. Sci. Rep.* **71**, 595-671 (2016).
2. Deng, X., et al. Surface chemistry of Cu in the presence of CO_2 and H_2O. *Langmuir* **24**, 9474-9478 (2008).
3. Garza, A. J., Bell, A. T. & Head-Gordon, M. Is Subsurface Oxygen Necessary for the Electrochemical Reduction of CO_2 on Copper? *J. Phys. Chem. Lett.* **9**, 601-606 (2018).
4. Bloch, J., Bottomley, D. J., Janz, S. & van Driel, H. M. Detection of subsurface oxygen on Cu(111): correlation of second-harmonic generation and Auger electron spectroscopy observations. *Surf. Sci.* **257**, 328-334 (1991).
5. Cavalca, F., et al. Nature and Distribution of Stable Subsurface Oxygen in Copper Electrodes During Electrochemical CO_2 Reduction. *J. Phys. Chem. C* **121**, 25003-25009 (2017).
6. Eilert, A., et al. Subsurface Oxygen in Oxide-Derived Copper Electrocatalysts for Carbon Dioxide Reduction. *J. Phys. Chem. Lett.* **8**, 285-290 (2017).
7. Bloch, J., Bottomley, D. J., Janz, S., Driel, H. M. v. & Timsit, R. S. Kinetics of oxygen adsorption, absorption, and desorption on the Cu(111) surface. *J. Chem. Phys.* **98**, 9167-9176 (1993).
8. Nagy, A. J., et al. The Correlation of Subsurface Oxygen Diffusion with Variations of Silver Morphology in the Silver–Oxygen System. *J. Catal.* **182**, 417-429 (1999).
9. Bao, X., Muhler, M., Pettinger, B., Schlögl, R. & Ertl, G. On the nature of the active state of silver during catalytic oxidation of methanol. *Catal. Lett.* **22**, 215-225 (1993).
10. Li, W.-X., Stampfl, C. & Scheffler, M. Subsurface oxygen and surface oxide formation at Ag(111): A density-functional theory investigation. *Phys. Rev. B* **67**, 045408 (2003).
11. Todorova, M., et al. Role of Subsurface Oxygen in Oxide Formation at Transition Metal Surfaces. *Phys. Rev. Lett.* **89**, 096103 (2002).
12. Li, W.-X., Stampfl, C. & Scheffler, M. Why is a Noble Metal Catalytically Active? The Role of the O-Ag Interaction in the Function of Silver as an Oxidation Catalyst. *Phys. Rev. Lett.* **90**, 256102 (2003).
13. Andryushechkin, B. V., Shevlyuga, V. M., Pavlova, T. V., Zhidomirov, G. M. & Eltsov, K. N. Adsorption of O_2 on Ag(111): Evidence of Local Oxide Formation. *Phys. Rev. Lett.* **117**, 056101 (2016).
14. Heine, C., Eren, B., Lechner, B. A. J. & Salmeron, M. A study of the O/Ag(111) system with scanning tunneling microscopy and x-ray photoelectron spectroscopy at ambient pressures. *Surf. Sci.* **652**, 51-57 (2016).
15. Campbell, C. T. & Paffett, M. T. The interactions of O_2, CO and CO_2 with Ag(110). *Surf. Sci.* **143**, 517-535 (1984).
16. Campbell, C. T. An XPS study of molecularly chemisorbed oxygen on Ag(111). *Surf. Sci.* **173**, L641-L646 (1986).
17. Campbell, C. T. Atomic and molecular oxygen adsorption on Ag(111). *Surf. Sci.* **157**, 43-60 (1985).
